# BA-12 Inhibits Angiogenesis via Glutathione Metabolism Activation

**DOI:** 10.3390/ijms20164062

**Published:** 2019-08-20

**Authors:** Herong Cui, Wenbo Guo, Beibei Zhang, Guoping Li, Tong Li, Yanyan Yuan, Na Zhang, Yuwei Yang, Wuwen Feng, Fuhao Chu, Shenglan Wang, Bing Xu, Penglong Wang, Haimin Lei

**Affiliations:** 1 School of Chinese Pharmacy, Beijing University of Chinese Medicine, Beijing 102488, China; 2 School of Pharmacy, Chengdu University of Traditional Chinese Medicine, Chengdu 610000, China; 3 School of Acupuncture and Massage, Beijing University of Chinese Medicine, Beijing 102488, China

**Keywords:** drug discovery, betulinic acid, ligustrazine, angiogenesis, metabolomics

## Abstract

There is a need for an efficient and low-cost leading compound discovery mode. However, drug development remains slow, expensive, and risky. Here, this manuscript proposes a leading compound discovery strategy based on a combination of traditional Chinese medicine (TCM) formulae and pharmacochemistry, using a ligustrazine–betulinic acid derivative (BA-12) in the treatment of angiogenesis as an example. Blocking angiogenesis to inhibit the growth and metastasis of solid tumors is currently one recognized therapy for cancer in the clinic. Firstly, based on a traditional *Prunella vulgaris* plaster, BA-12 was synthesized according to our previous study, as it exhibited better antitumor activities than other derivatives on human bladder carcinoma cells (T24); it was then uploaded for target prediction. Secondly, the efficacy and biotoxicity of BA-12 on angiogenesis were evaluated using human umbilical vein endothelial cells (HUVECs), a quail chick chorioallantoic membrane, and *Caenorhabditis elegans*. According to the prediction results, the main mechanisms of BA-12 were metabolic pathways. Thus, multiple metabolomics approaches were applied to reveal the mechanisms of BA-12. Finally, the predictive mechanisms of BA-12 on glutathione metabolism and glycerophospholipid metabolism activation were validated using targeted metabolomics and pharmacological assays. This strategy may provide a reference for highly efficient drug discovery, with the aim of sharing TCM wisdom for unmet clinical needs.

## 1. Introduction

Despite many advances in recent decades, drug development remains slow, expensive, and risky [1]. Screening a small molecule to become a drug candidate typically takes 4–5 years, costs $14–25 million, and has a loss rate of more than 50%. Recent estimates show a loss rate of up to 97% from drug candidates to commercialized products, with total costs of drug development (including loss costs and capital costs) exceeding $2.6 billion [2]. The selection of biological targets is a key source of loss [3]. As a result, new drug discovery centers around the world invested heavily in translational science to more effectively validate the role of biological targets in human disease and identify the most appropriate patient populations for evaluating potential drugs [4]. In the search for effective treatments, new models of drug development were successfully developed, most notably antibody-based therapies [5]. However, some targets with a strong biological basis are very challenging for the current small-molecule drug discovery technology [6,7]. Therefore, it is urgent to establish an efficient and low-cost drug discovery and design mode.

Fast-growing tumors are usually starved of blood, which can stimulate angiogenesis [8,9,10]. Angiogenesis refers to the formation of vessels on the basis of existing vessels via the sprouting, proliferation, and migration of endothelial cells [11,12,13,14]. In the process of tumor growth, oxygen supply, and metastasis, the further growth of malignant solid tumors must rely on angiogenesis for breaking through the epithelial basement membrane. Thus, blocking angiogenesis and then inhibiting the growth and metastasis of solid tumors is currently the recognized therapy for tumors in the clinic [15,16,17]. However, some tumors such as pancreatic adenocarcinoma, prostate cancer, and melanoma are resistant, with modest improvements in progression-free survival [18,19,20,21,22]. It is crucial to develop anti-angiogenesis agents with sufficient research on the involved mechanisms.

Through a long-term clinical practice, the theory of compatibility with traditional Chinese medicine (TCM) was formed, which may provide new ideas for drug discovery. We made many attempts to combine effective components based on the compatibility theory, in order to efficiently screen leading compounds, and we obtained one high-efficiency, low-toxicity, and highly selective anticancer leading compound with a value of nearly $100 million [23]. Here, this manuscript tries to propose this drug discovery strategy based on the TCM compatibility theory and the combination principle, taking an effective ligustrazine–betulinic acid derivative (BA-12) as an example. Furthermore, the method of metabolomics [24,25] was used to dissect the mechanisms more quickly and systematically, providing guidance for the rational development of medication using this agent.

**Figure 1:**
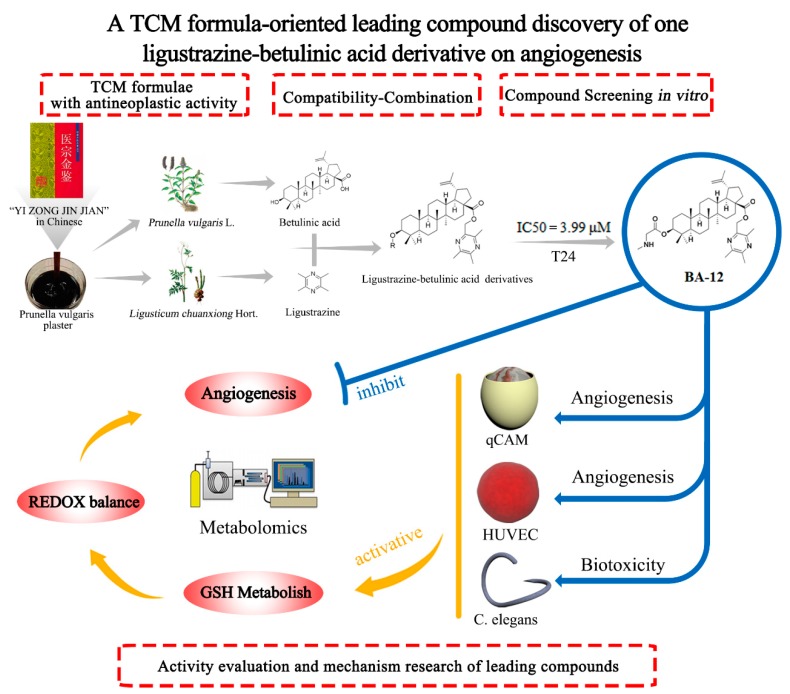
The leading compound discovery strategy based on the compatibility theory of traditional Chinese medicine and the combination principle of pharmacochemistry.

This leading compound discovery strategy is depicted in Figure 1. Firstly, based on a traditional *Prunella vulgaris* plaster, BA-12 was synthesized via a combination of ligustrazine with betulinic acid according to our previous study [23], as it exhibited better antitumor activities than other derivatives on human bladder carcinoma cells (T24); it was then uploaded to PharmMapper server for target prediction [26,27]. Then, the efficacy of BA-12 on angiogenesis was evaluated using a quail chick chorioallantoic membrane (qCAM) and human umbilical vein endothelial cells (HUVECs), and its biotoxicity was evaluated using *Caenorhabditis elegans* (*C. elegans*). According to the prediction results, the main mechanisms of BA-12 were metabolic pathways. Thus, GC–MS-based and UPLC–TOF-MS-based untargeted metabolomics approaches, together with pathway analysis, were applied to reveal the mechanisms of BA-12 via a systematic analysis of specific biomarkers and biochemical pathways conducted with multivariate data analysis methods. Finally, the predictive mechanisms of BA-12 on glutathione metabolism and glycerophospholipid metabolism activation were validated using targeted metabolomics and pharmacological assays. The strategy in this manuscript may provide a reference for current drug discovery efforts.

## 2. Results

### 2.1. Synthesis, Chemical Information, and Antitumor Activities of BA-12

**Figure 2:**
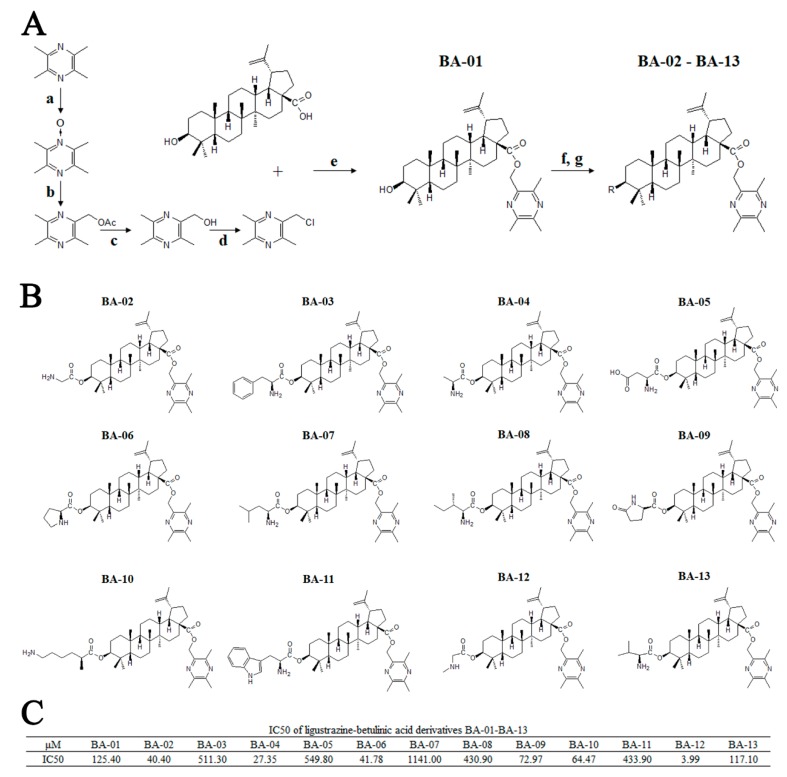
Synthesis, chemical information, and antitumor activities of ligustrazine–betulinic acid derivatives (BA-01–BA-13) on human bladder carcinoma (T24) cells. (**A**) Synthesis of ligustrazine–betulinic acid derivatives (BA-01–BA-13). Conditions and reagents: (a) acetic acid and 30% H_2_O_2_, reflux at 90 °C for 6 h; (b) acetic anhydride, reflux at 105 °C for 2 h; (c) methanol (MeOH):tetrahydrofuran (THF):H_2_O ¼ 1:3:1 with NaOH for 1 h; (d) tosyl chloride (TsCl), THF, 4-dimethylaminopyridine (DMAP), trimethylamine (TEA) for 12 h; (e) dry K_2_CO_3_, dry *N*,*N*-dimethylformamide (DMF) at 25 °C for 12 h; (f) *N*-benzyloxycarbonyl (*N*-Cbz) or *N*-butyloxy carbonyl (*N*-Boc) amino acids, DMAP, 1-ethyl-3-(3-dimethylaminopropyl)carbodiimide hydrochloride (EDCI), dichloromethane (DCM) at 25 °C for 12 h; (g) Pd/C (10%), MeOH at 25°C for 12 h, or trifluoroacetic acid (TFA) in dry DCM at 0 °C for 2 h. (**B**) Structures of ligustrazine–betulinic acid derivatives (BA-01–BA-13). (**C**) Half maximal inhibitory concentration (IC_50_) values of ligustrazine–betulinic acid derivatives (BA-01–BA-13) on T24 cells, evaluated using 3-(4,5-dimethylthiazol-2-yl)-2,5-diphenyltetrazolium bromide (MTT) assays.

The ligustrazine–betulinic acid derivatives (BA-01–BA-13) were synthesized according to our previous study [23]. Firstly, in order to yield 2-(chloromethyl)-3,5,6-trimethylpyrazine, (3,5,6-trimethylpyrazin-2-yl)methanol was reacted with tosyl chloride (TsCl) in 4-dimethylaminopyridine (DMAP), trimethylamine (TEA), and tetrahydrofuran (THF). Then, it was reacted with betulinic acid in *N*,*N*-dimethylformamide (DMF) to obtain BA-01. The derivatives of BA-02–BA-13 were obtained from the corresponding protected (*N*-butyloxy carbonyl (*N*-Boc), *N*-benzyloxycarbonyl (*N*-Cbz)) amino acids and BA-01 via esterification with 1-ethyl-3-(3-dimethylaminopropyl)carbodiimide hydrochloride (EDCI) (Figure 2A). The structures are shown in Figure 2B. The in vitro antitumor activity of these derivatives was evaluated on T24 cells using the 3-(4,5-dimethylthiazol-2-yl)-2,5-diphenyltetrazolium bromide (MTT) assay, and the half maximal inhibitory concentration (IC_50_) values were shown as Figure 2C.

**Figure 3:**
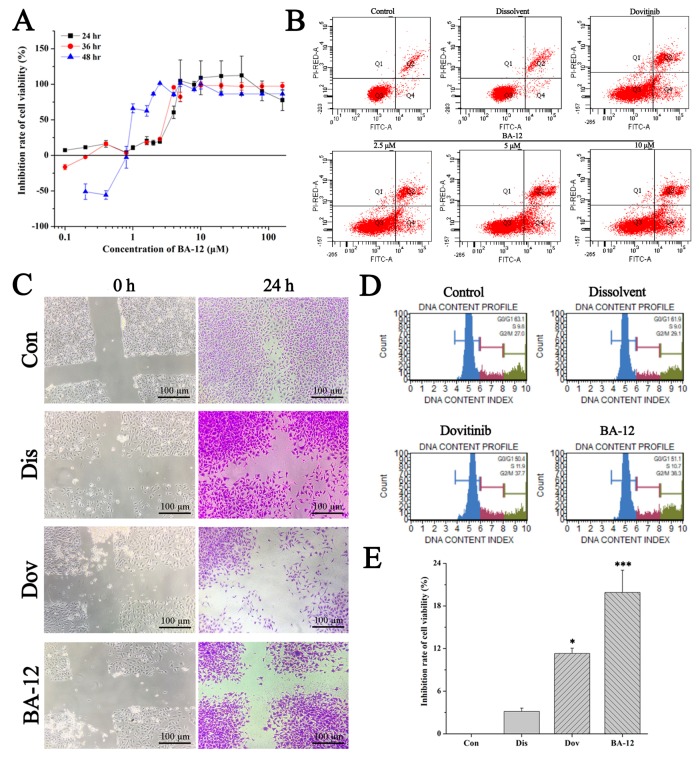
The in vitro antitumor activity of BA-12 on T24 cells. (**A**) Inhibition rate of cell viability of T24 cells for MTT assays (cells treated with BA-12 at doses of 0.25–160 μM) for 24, 48, and 72 h. (**B**) Apoptosis analysis of T24 cells induced by agents using AnnexinV-fluorescein isothiocyanate (FITC)/propidium iodide (PI) staining. (**C**) Results for wound scratch assay (cells treated with BA-12 at doses of 2.5 μM) after 24 h under the microscope (100×). The most representative fields are shown. (**D**) Cell cycle analysis using PI staining (cells treated with BA-12 at doses of 2.5 μM). (**E**) Inhibition rate of cell viability of T24 cells for MTT assays (cells treated with BA-12 at doses of 2.5 μM) for 24 h. ANOVA with a post hoc test was used to calculate the significance of the differences; * *p* < 0.05, *** *p* < 0.001 compared with the dissolvent group. Experiments were executed three times. Results are displayed as means ± SD.

**Figure 4:**
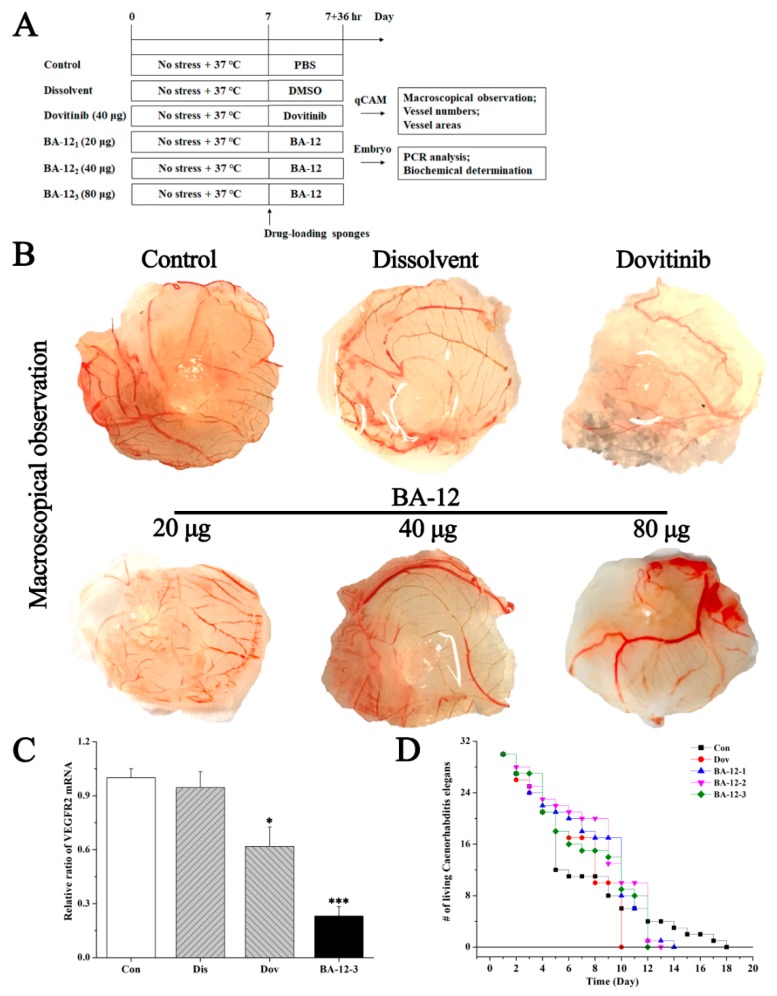
The effect of BA-12 on angiogenesis using a quail chick chorioallantoic membrane (qCAM) and human umbilical vein endothelial cells (HUVECs), and its biotoxicity based on a survival essay with *Caenorhabditis elegans*. (**A**) Schematic diagram of the experimental design. Con, control group treated with phosphate-buffered saline (PBS); Dis, dissolvent group treated with dissolvent containing 0.5% dimethyl sulfoxide (DMSO); Pos, positive control group treated with dovitinib (40 μg); BA-12 1–3, groups treated with BA-12 at doses of 20, 40, and 80 μg, respectively. (**B**) Inhibition of angiogenesis by BA-12 for qCAM assays in a dose-dependent manner. (**C**) Messenger RNA (mRNA) levels of vascular endothelial growth factor receptor 2 (VEGFR2) in qCAM samples. The most representative fields are shown. (**D**) Survival assay using *Caenorhabditis elegans* for biotoxicity detection. ANOVA with a post hoc test was used to calculate the significance of the differences; * *p* < 0.05, *** *p* < 0.001 compared with the dissolvent group. Experiments were executed three times. Results are displayed as means ± SD.

Among these, BA-12 exhibited better antitumor activities as indicated by a significant (*p* < 0.05) increase in apoptosis, and a significant (*p* < 0.05) decrease in proliferation and migration of T24 cells (Figure 3). A certain dose (2.5 μM) of dovitinib was set as a positive control in this part of our experiment [28,29]. The MTT assay showed the effect of BA-12 on T24 cells (Figure 3A). Then, the effects of BA-12 on apoptosis in T24 cells were further determined by flow cytometric analysis (Figure 3B and Table 1). The apoptosis ratios increased to 20.3% (2.5 μM), 31.0% (5 μM), and 33.3% (10 μM), while that of the control was 5.7%, indicating that BA-12 could induce T24 cell apoptosis in a concentration-dependent manner. Thus, 2.5 μM BA-12 was chosen for subsequent studies. As the data show, 2.5 μM BA-12 could significantly (*p* < 0.05) inhibit cell viability, scratch healing percentage, and cell cycle compared to the dissolvent group (Figure 4C–E and Table 1). These results revealed the significant (*p* < 0.05) inhibitory effect of BA-12 on T24 cells.

Then, the chemical information of BA-12 was further confirmed by UPLC–MS in the positive electrospray ionization (ESI+) mode (Supplementary Materials Figure S1), and the prediction pathways of BA-12 were determined using PharmMapper, as shown in Supplementary Materials Table S4.

### 2.2. BA-12 Inhibits Angiogenesis in a Dose-Dependent Manner with Lower Biotoxicity

The efficacy of BA-12 on angiogenesis was firstly evaluated on qCAM assays, and the experimental design is shown in Figure 4A. Dovitinib was set as a positive control in this part of our experiment [28]. Amounts of 20, 40, and 80 μg of BA-12 were chosen according to our preliminary tests. Macroscopical observation revealed the inhibitory effect of BA-12 and dovitinib on angiogenesis compared to the dissolvent group in a dose-dependent manner, and 40 μg of BA-12 may play a significant (*p* < 0.05) inhibitory role with a decrease in vessel number and vessel area (Figure 4B and Supplementary Materials Figure S3). Furthermore, PCR analysis showed that BA-12 resulted in a significant (*p* < 0.05) decrease in vascular endothelial growth factor receptor 2 (VEGFR2) messenger RNA (mRNA) within 36 h (Figure 3C). Moreover, the results of desorption electrospray ionization mass spectrometry (DESI-MS) imaging of qCAMs are depicted in Figure S2 and Supplementary Materials Table S1 for quality control of the objects in this research.

Furthermore, biotoxicity was preliminarily evaluated by the survival assay of *C. elegans*. By comparison of the positive control group (40 μg dovitinib), 20, 40, and 80 μg of BA-12 exerted lower biotoxicity based on a relatively slower death rate (Figure 4D and Supplementary Materials Figure S9). 

### 2.3. BA-12 Inhibits Proliferation, Migration, and Tube Formation of HUVEC Cells

**Figure 5:**
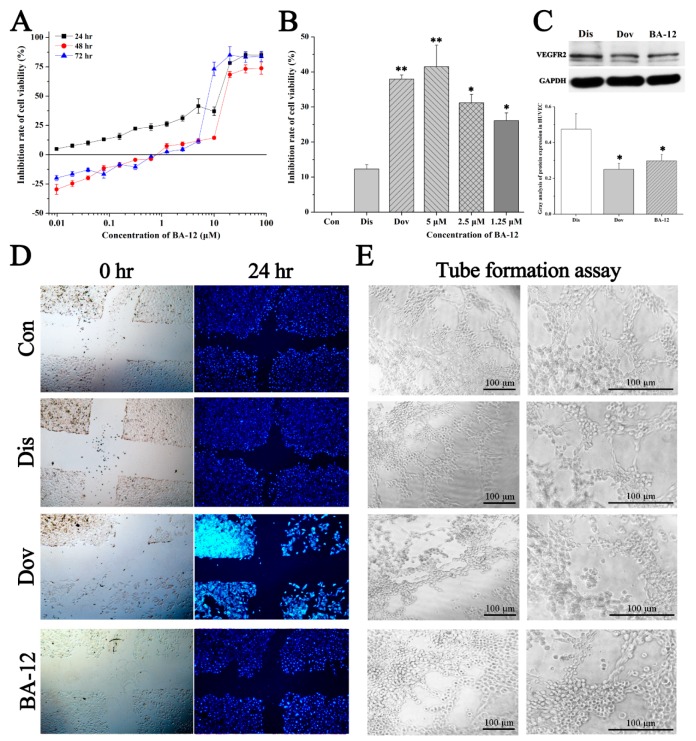
The effect of BA-12 on angiogenesis in HUVECs. (**A**) Inhibition rate of cell viability of HUVECs by MTT assays (cells treated with BA-12 at doses of 0.25–160 μM) for 24, 48, and 72 h. (**B**) Inhibition rate of cell viability of HUVECs for MTT assays for 24 h. (**C**) Cellular VEGFR levels and grayscale analysis. (**D**,**E**) Results for wound scratch assay and tube formation assay after 24 h under the microscope (100×). Con, control group treated with PBS; Dis, dissolvent group treated with dissolvent containing 0.5% DMSO; Pos, positive control group treated with dovitinib (2.5 μM); BA-12, groups treated with BA-12 at doses of 2.5 μM. Representative fields are shown. ANOVA with a post hoc test was applied for the significance of the differences; * *p* < 0.05, ** *p* < 0.01 compared with the dissolvent group. Experiments were executed three times. Results are displayed as means ± SD.

Subsequently, the efficacy of BA-12 on angiogenesis was evaluated based on a proliferation, migration, and tube formation assay with HUVECs. A certain dose (2.5 μM) of dovitinib was set as a positive control in this part of our experiment. Then, 2.5 μM BA-12 was chosen for subsequent studies according to the MTT assay (Figure 5A). As shown by the results, 2.5 μM BA-12 could significantly (*p* < 0.05) inhibit cell viability, scratch healing percentage, and the branch points of tubes compared to the control group (Figure 5B–E and Supplementary Materials Figure S4). These results revealed the inhibitory effect of BA-12 on the proliferation, migration, and tube formation of HUVECs. Furthermore, the effect of BA-12 on angiogenesis was re-evaluated by Western blotting. As a result (Figure 5C and Supplementary Materials Figure S10), the protein expression of VEGFR2 decreased significantly (*p* < 0.05) due to 2.5 μM BA-12 within 24 h compared to the control group, verifying that 2.5 μM BA-12 could inhibit the angiogenesis of HUVECs.

### 2.4. Untargeted Metabolomics Reveals Altered Metabolic Pathways Regulated by BA-12

**Figure 6:**
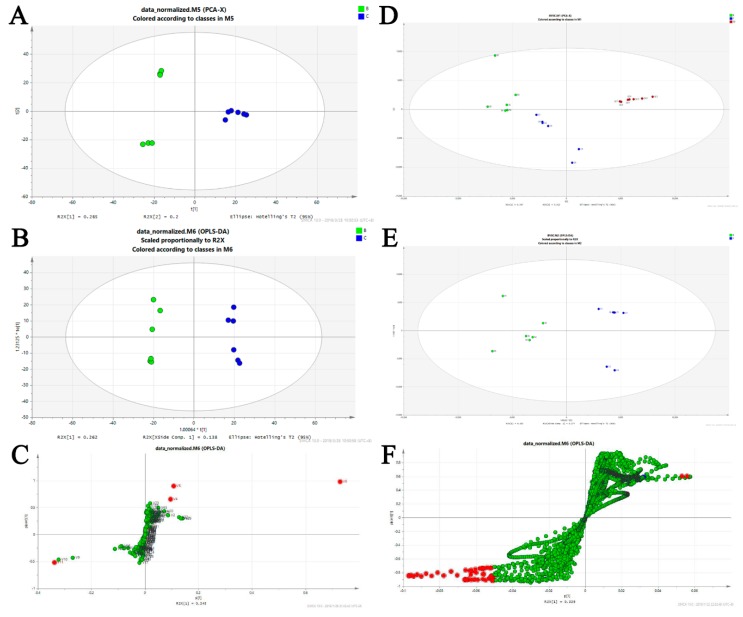
Biomarker identification and altered metabolic pathways regulated by BA-12. For GC–MS metabolomics: (**A**) score plots of control (C group, PBS) and BA-12 (B group, 2.5 μM) from principal component analysis (PCA) for principal component 1 (PC1) versus PC2; (**B**) score plots of control (C group, PBS) and BA-12 (B group, 2.5 μM) from orthogonal projections to latent structures discriminant analysis (OPLS-DA) model for the pairwise comparisons; (**C**) S-plot of the OPLS-DA model for the B and C groups. The points in red indicate the identified biomarkers. For UPLC–TOF-MS metabolomics: (**D**) score plots of control (C group, PBS) and BA-12 (B group, 2.5 μM) from PCA in the positive electrospray ionization (ESI+) mode for PC1 versus PC2; (**E**) score plots of control (C group, PBS) and BA-12 (B group, 2.5 μM) from OPLS-DA model for the pairwise comparisons; (**F**) S-plot of the OPLS-DA model for the B and C groups. The points in red indicate the identified biomarkers.

According to the prediction results of BA-12, the main mechanisms of BA-12 were metabolic pathways (Supplementary Materials Table S4). Thus, multiple metabolomics approaches were applied to reveal the mechanisms of BA-12. GC–MS-based untargeted metabolomics was firstly executed (Supplementary Materials Figures S5 and S6) for the score plots of principal component analysis (PCA) and orthogonal projections to latent structures discriminant analysis (OPLS-DA) models (Figure 6A,B). The score plot of the model with an *R*^2^*Y* (cum) of 0.993 and *Q*^2^ (cum) of 0.873 showed good fitness and good predictive ability (Supplementary Materials Table S2 and Figures S7 and S8). The variable importance for projection (VIP) values and S-plots of the OPLS-DA model (Figure 6C) were determined for the variables showing a high correlation with group separation. Specifically, variables with the condition of |*p* (corr)| ≥ 0.50 and VIP value >1 were screened as key metabolites (candidate biomarkers). Then, key metabolites were identified by the online METLIN database and the National Institute of Standards (NIST14) mass spectral libraries. A total of four potential biomarkers were identified, as shown in Table 2. MetaboAnalyst was applied for the pathway analysis. Results (Table 3) showed that four pathways (*p* < 0.05) including glutathione (GSH) metabolism (most significant), pyrimidine metabolism, arginine and proline metabolism, and tryptophan metabolism were affected by BA-12. 

Subsequently, UPLC–TOF-MS-based untargeted metabolomics was executed (Supplementary Materials Figure S8) for the score plots of PCA and OPLS-DA models (Figure 6D,E). The score plot of the model with an *R*^2^*Y* (cum) of 0.999 and *Q*^2^ (cum) of 0.992 showed good fitness and good predictive ability (Supplementary Materials Table S3). The variable importance for projection (VIP) values and S-plots of the OPLS-DA model (Figure 6F) were determined for the variables showing a high correlation with group separation. Specifically, variables with the condition of |*p* (corr)| ≥ 0.50 and VIP value >1 were screened as key metabolites (candidate biomarkers). Then, key metabolites were identified by the online METLIN database. A total of four potential biomarkers were identified, as shown in Table 4. MetaboAnalyst was applied for the pathway analysis. Results showed that glycerophospholipid metabolism (*p* < 0.05) was affected by BA-12 (Table 5).

### 2.5. Mechanism Validation of BA-12 on Glutathione Metabolism and Glycerophospholipid Metabolism Activation

**Figure 7:**
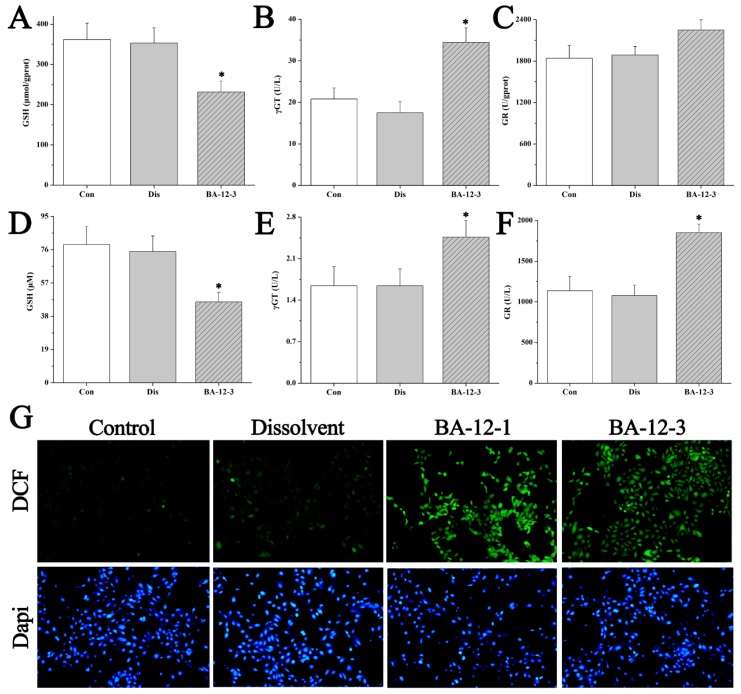
Mechanisms of BA-12 for glutathione (GSH) metabolism activation. (**A**–**C**) GSH, γ-glutamyl transferase (γGT), and glutathione reductase (GR) levels in quail samples, respectively. (**D**–**F**) GSH, γGT, and GR levels in cell samples, respectively. (**G**) Morphological observation and relative fluorescence intensity of HUVECs induced by BA-12 (10, 2.5 μM) for 24 h by reactive oxygen species (ROS) assay and 4′,6-diamidino-2-phenylindole (DAPI) staining under the fluorescence microscope (100×). Dichlorofluorescein (DCF), compounds with fluorescence for the reflection of ROS levels. The most representative fields are shown. Con, control group treated with PBS; Dis, dissolvent group treated with dissolvent containing 0.5% DMSO; BA-12-1 and BA-12-3, groups treated with BA-12 at doses of 5 and 1.625 μg, respectively. ANOVA with a post hoc test was applied for the significance of the differences; * *p* < 0.05 compared with the dissolvent group. Experiments were executed three times. Results are displayed as means ± SD.

**Figure 8:**
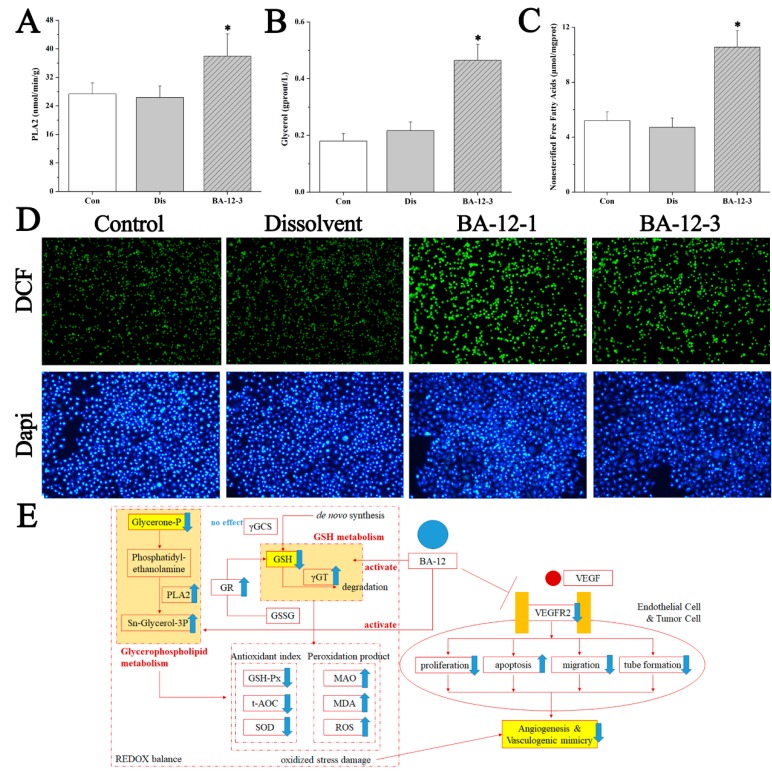
Mechanisms of BA-12 for glycerophospholipid metabolism activation. (**A**–**C**) Phospholipase A2 (PLA2), glycerol, and non-esterified free fatty acid (NEFA) levels in quail samples, respectively. (**D**) Morphological observation and relative fluorescence intensity of T24 cells induced by BA-12 for 24 h by ROS assay and DAPI staining under the fluorescence microscope (100×). DCF, compounds with fluorescence for the reflection of ROS levels. The most representative fields are shown. (**E**) Schematic diagram of the efficacy and mechanism of BA-12 for angiogenesis. Con, control group treated with PBS; Dis, dissolvent group treated with dissolvent containing 0.5% DMSO; BA-12-1 and BA-12-3, groups treated with BA-12 at doses of 5 and 1.625 μg, respectively. ANOVA with a post hoc test was applied for the significance of the differences; * *p* < 0.05 compared with the dissolvent group. Experiments were executed three times. Results are displayed as means ± SD.

To validate the main mechanism of BA-12, the indicators correlated with glutathione metabolism and glycerophospholipid metabolism were measured by UPLC–QTOF-MS-based targeted metabolomics and pharmacological assays. The results (Figure 7 and Figure 8, Table 6, and Supplementary Materials Figure S8) showed that the content of GSH and glycerophospholipid in both quail and cell samples was significantly (*p* < 0.05) lower than in the dissolvent group. Furthermore, the activity of γ-glutamyl transferase (γ-GT), glutathione reductase (GR), and phospholipase A2 (PLA2), and the level of glycerol and non-esterified free fatty acids were significantly (*p* < 0.05) higher, whereas the activity of γ-glutamylcysteine synthetase (γ-GCS) was not significantly (*p* < 0.05) changed. These findings indicate that BA-12 resulted in the activation of GSH metabolism and glycerophospholipid metabolism, with the subsequent degradation of GSH and glycerophospholipids. This may further impact the redox balance, as indicated by the significant (*p* < 0.05) increase in peroxidation products such as monoamine oxidase (MAO), malondialdehyde (MDA), and reactive oxygen species (ROS), and the significant (*p* < 0.05) decrease in antioxidant indexes such as glutathione peroxidase (GSH-Px), total antioxidant capacity (t-AOC), and superoxide dismutase (SOD) (Supplementary Materials Figure S11). These results suggest the efficacy of BA-12 on GSH and glycerophospholipid metabolism, as well as REDOX balance, leading to the inhibition of angiogenesis.

## 3. Discussion

Despite the completion of the human genome project, there are still many complex diseases that are hard for humans to cure [29]. Drug discovery is once again the focus of the international pharmaceutical field, and it is common practice to analyze the interactions between drugs dependent on computational chemistry [30]. Yet, the loss rate for screening a computational molecule as a drug candidate is about 50% or higher [4]. Therefore, it is urgent to establish an efficient and low-cost drug discovery and design mode. The drug discovery strategy described in this manuscript (Figure 1) will bring new opportunities for modern drug research and development.

The quail chick chorioallantoic membrane (qCAM) model is an optimized experimental model for evaluating angiogenesis based on the CAM model published in Science [31], with advantages of an intuitional, objective, rapid, simple, and low-cost design. The qCAM model generally selects live quail embryos incubated on the sixth to seventh day for experimental research. The histological structure of the qCAM contains three layers: ectoderm (below the shell membrane, consisting of the chorionic epithelium), mesoderm (connective tissue rich in capillaries), and the endoderm (in the allantoic sac and formed by the allantoic membrane endothelium). With the increase of embryo age, the capillaries became more and more abundant. The multi-factor parallel experiments of numerous groups verified that angiogenesis of a leading compound can be evaluated relatively reliably [31,32,33,34]. Transcriptome sequencing of quail was completed in 2013 [35], and this provides the possibility for mRNA detection of the qCAM model for further research. As a result (Figure 4 and Figure 5), we revealed the effect of BA-12 on angiogenesis based on the qCAM model and HUVEC assays, while further studies are needed to find its targets.

It is crucial to develop drugs targeting angiogenesis with sufficient mechanisms, in order to predict which patients will and will not benefit before the initiation of therapy. Large doses of reactive oxygen species (ROS) can induce oxidative stress and lead to oxidative damage [36]. Antioxidant defense systems includes glutathione reductase (GR), superoxide dismutase (SOD), and so on. GR can regenerate GSH and catalyze the reduction of oxidized glutathione (GSSG) to glutathione (GSH). SOD can catalyze the production of O^2−^. When oxidative damage happens, antioxidant enzymes such as GR and SOD play a great role [37]. Furthermore, the imbalance of oxidative stress caused by the activation of GSH metabolism leads to the abnormality of glycerophospholipid metabolism. In this manuscript, GSH metabolism activation induced by BA-12 could result in the functional alteration of the antioxidant defense system of HUVECs (Figure 7 and Figure 8, Table 6, and Supplementary Materials Figure S8). These findings (Figure 8E) indicate that GSH metabolism activation induced by BA-12 can induce excessive ROS production, and affect the antioxidant defense system and glycerophospholipid metabolism, thereby inhibiting angiogenesis [38]. These effects of BA-12 may be associated with the efficacy of drug resistance [38,39,40] and the regulation of gut microbiota [41,42]; thus, further studies are needed.

## 4. Materials and Methods

### 4.1. Chemicals and Cell Culture Materials

HUVEC and T24 cell lines were obtained from the Institute of Peking Union Medical College. The cell lines were characterized by Genetic Testing Biotechnology Corporation (Genetic Testing Biotechnology Corporation, Suzhou, China) using short tandem repeat (STR) markers. Dulbecco’s modified Eagle’s medium (DMEM), and heat-inactivated fetal bovine serum (FBS) were obtained from GIBCO Invitrogen (GIBCO Invitrogen, Barcelona, Spain). Streptomycin and penicillin and were obtained from Thermo Scientific (Waltham, USA). Black and yellow quail eggs were provided by the Qingfeng Aichongyuan livestock farm, Shandong Rizhao, China. The compounds 2-chloro-l-phenylalanine (≥98%) and *N*,*O*-bis(trimethylsilyl)trifluoroacetamide with 1% trimethylchlorosilane (BSTFA + 1% TMCS) were obtained from Shanghai Macklin Biochemical Co., Ltd. (Shanghai Macklin Biochemical Co., Ltd., Shanghai, China; CAS number 103616-89-3, 25561-30-2). Methoxyamine hydrochloride (≥98%) and thiazolyl blue tetrazolium bromide (MTT, ≥98%) were obtained from Sigma-Aldrich (Sigma-Aldrich, St. Louis, Missouri, USA; CAS number 593-56-6). Dovitinib (Master of Small Molecules, Shanghai, China; CAS No. 405169-16-6, Lot number HY-50905, 99.31%) was purchased from Master of Small Molecules, China. Methanol (≥99.9%) and pyridine (≥99%) were obtained from VWR (VWR, Leuven, Belgium). Power SYBR Green PCR Master Mix reagents were provided by Life Technologies (Thermo Fisher Scientific Inc.). Methanol and acetonitrile and (HPLC grade) were purchased from Burdick and Jackson (Burdick and Jackson, Ulsan, Korea) and Merck (Merck, Darmstadt, Germany), respectively. Double-distilled water was purified by a Millipore water purification system (Millipore, Bedford, MA, USA). All other chemicals used were of analytical grade.

### 4.2. Quail Chick Chorioallantoic Membrane (qCAM) Assay

The quail chick chorioallantoic membrane (qCAM) assay was performed taking Labastie’s method [43] for reference as an ex vivo model to assess the potential of BA-12 to inhibit vascularization. Briefly, the eggs were incubated at 37 °C, 65% relative humidity in an egg incubator. On day seven, drug-loading sponges (5 mm diameter; *n* = 6) of BA-12 (20, 40, and 80 μg) were sterilized and placed over qCAMs and, later, the eggshell was sealed. On day nine, the window was carefully dissected, and qCAMs were photographed in situ. Three blind observers counted the number of blood vessels approaching the sponges. Furthermore, membranes were retrieved carefully with forceps as samples. The six biological replicates in their respective groups were analyzed with a single technical replicate.

### 4.3. RNA Isolation and Real-Time PCR Analysis

Total RNA was isolated by TRIzol reagent (Invitrogen, Thermo Fisher Scientific Inc., Sunnyvale, CA, USA). For qRT-PCR, the first-strand complementary DNA (cDNA) was synthesized using an RA First-Strand cDNA Synthesis Kit (Thermo Fisher Scientific Inc., Sunnyvale, CA, USA) and performed with an ABI Prism 7500 sequence detection system. The specific primers for VEGFR2 and Actin were purchased from Life Technologies (Life Technologies, Thermo Fisher Scientific Inc., Sunnyvale, CA, USA). Data analysis was performed using the 2^−ΔΔCT^ method. Actin expression was used to normalize the obtained data. Primer sequences for quail VEGFR2 primers were 5′–AGCATAGACAGCCCTTTGGT–3′ (forward primer) and 5′–CACAATCTCTGCTGGTGCAA–3′ (reverse primer), while those for quail Actin primers were 5′–CTGGCACCTAGCACAATGAA–3′ (forward primer) and 5′–CTGCTTGCTGATCCACATCT–3′ (reverse primer). The six biological replicates were analyzed with a single technical replicate.

### 4.4. Survival Assay

*C. elegans* was used for the survival assay in a primary toxicity test. Synchronized L1 nematodes were cultured in S-medium containing *Escherichia coli* NA22 until L4. Afterward, 5-fluoro-2’-deoxyuridine (FUdR; Sigma, St. Louis, MO, USA) with a concentration of 75 μg/mL was applied to prevent self-fertilization. After incubation for 24 h, these nematodes were transferred into 96-well plates. The living and dead nematodes (~30 for each treatment) were scored microscopically every 12 h until all dead. The six biological replicates were analyzed with a single technical replicate.

### 4.5. Cell Viability Assay

The MTT assay was performed as follows [44]: T24 or HUVEC cells were cultured in 96-well plates. Then, BA-12 in a concentration range of 0.25–160 μM was added. The lethal concentration of the cells was estimated according to the MTT reduction assay. The optical density of each well was measured by a BIORAD 550 spectrophotometer plate reader at 550 nm.

### 4.6. Apoptosis Analysis by Flow Cytometric Using Annexin V-Fluorescein Isothiocyanate (FITC)/Propidium Iodide (PI) Staining

The T24 cells were seed in six-well plates and then the agents were added. After 72 h, all cells were collected, washed twice, and centrifuged at 1000 rpm for 5 min. Cells were resuspended in binding buffer containing Annexin V-FITC and PI. Then, cells were analyzed with a flow cytometer after avoiding a light reaction.

### 4.7. Wound Scratch Assay

T24 or HUVEC cells were cultured in 24-well plates until forming a monolayer; then, a scratch was generated by a 10-μL pipette tip. T24 cells were allowed to migrate for 24 h. Images of the same location were taken using light microscopy (10×, Nikon Eclipse TiE, Tokyo, Japan), and the area of gap closure was calculated by Image-Pro Plus software (version 5.0, National Institutes of Health, Bethesda, MD, USA).

### 4.8. Cell Cycle Analysis Using PI Staining

T24 cells were seeded in 12-well plates and BA-12 was added for 24 h. Then, cells were collected, washed twice with cold phosphate-buffered saline (PBS), and centrifuged at 1000 rpm for 5 min. Subsequently, 70% cold ethanol was added for 12 h at 4 °C. Cells were washed with PBS, and stained with propidium iodide for 30 min at 37 °C avoiding light. Then, red fluorescence was detected by flow cytometry.

### 4.9. Tube Formation Assay

The tube formation assay was executed by assessing the formation of tube networks as described before [45]. Firstly, HUVECs were seeded in 96-well plates with a basement membrane matrix (Corning Matrigel, New York, NY, USA; CAS number 356234) coated with 10,000 cells per well. Then, BA-12 was added following a 6-h incubation. Tube networks were assessed by light microscopy (10×, Nikon Eclipse TiE) in at least three random fields, and the number of branch points in tube networks was quantified by Image-Pro Plus software (version 5.0, National Institutes of Health, Bethesda, MD, USA).

### 4.10. Western Blotting

The cell samples were ultrasonically treated in lysis buffer on ice, and the supernatant was collected after 12,000 rpm centrifugation for 10 min at 4 °C. The protein concentration was determined by a bicinchoninic acid (BCA) protein assay kit. Proteins were loaded onto an SDS-polyacrylamide gel electrophoresis (PAGE) gel, and then transferred to a polyvinylidene fluoride (PVDF) membrane. The PVDF membrane was blocked with blocking buffer for 2 h at room temperature, and incubated with specific primary antibodies against VEGFR2 (Servicebio, Shanghai, China; GB11190) and glyceraldehyde 3-phosphate dehydrogenase (GAPDH; Servicebio, Shanghai, China; GB12002) overnight at 4 °C. Next, membranes were incubated with secondary horse radish peroxidase (HRP)-conjugated immunoglobulin G (IgG) antibody (Servicebio, Shanghai, China; GB23303) for another 1 h at room temperature after washing. PVDF membranes were exposed using a Tanon 4200SF Chemiluminescent Imaging System (Shanghai Tanon Science & Technology Co., Ltd., Shanghai, China).

### 4.11. GC–MS-Based Untargeted Metabolomics Analysis for Cell Samples

#### 4.11.1. Sample Collection and Preparation [44]

T24 cells were centrifuged and washed with PBS. an ice-cold methanol:water solution (70:30, *v*/*v*) was added and scraped. Cells were sonicated and centrifuged for 10 min at 3000× *g* at 4 °C. The supernatant was collected for intracellular fingerprints. Quality control samples (QCs) were prepared as a pool of samples. Samples were kept at −80 °C until analysis.

Samples were added with 20 μL of 2-chloro-l-phenylalanine (0.3 mg/mL) as an internal standard, mixed in a vortex, and evaporated to dryness at room temperature. The derivatization process was carried out. Samples were added to a methoxyamine solution, incubated at 70 °C for 60 min, and BSTFA with 1% TMCS was added, before being incubated at room temperature for 60 min. Then, the samples dissolved in *n*-hexane were transferred to vials for GC–MS analysis. The six biological replicates were analyzed with a single technical replicate.

#### 4.11.2. Gas Chromatography and Mass Spectrometry Settings

The chromatographic analysis was performed using an Agilent 7890B/5977 GC–MS system (Agilent Technologies, Santa Clara, CA, USA), with a 5MS capillary column (30 m × 0.25 mm × 0.25 μm); the carrier gas was helium C-60 at a constant flow rate of 1.0 mL/min. Then, samples (2 μL) were injected in split mode (ratio 1:20). The injector temperature was 250 °C (held for 20 min). Total separation run time was 26 min; the oven temperature was set at 70 °C for 2 min, increasing to 250 °C (rate 15 °C/min), held for 2 min, increasing to 300 °C (rate 10 °C/min), and held for 5 min. The MS detector was operated in electron ionization (EI) mode (70 eV), and the EI temperature was 150 °C. Full scan mode was set for data acquisition with a mass range between 50 and 500 *m*/*z*. QC samples were repeatedly injected under the same conditions.

#### 4.11.3. Data Processing and Metabolic Pathway Analysis

Data were processed with PiroTrans (GL Science Inc. Tokyo, Japan) and LineUp (Infometrix Inc., Bothell, WA, USA). Peaks were rejected with signal-to-noise (S/N) ratios lower than 10. Data filtering, data normalization, missing value estimation, and fold changes were obtained using the MetaboAlalyst 3.0 online software. Resultant data matrices were analyzed by the SIMCA-P+ 13.0 (Umetrics, Umeå, Sweden) software. The significant differences were screened using the fold change value (>1.5) combined with the *t*-test (*p* < 0.05) and ANOVA (*p* < 0.05). Variables with significant changes were determined as potential biomarkers for further identification of molecular formulas. Biomarkers were tentatively identified by the online METLIN database and the National Institute of Standards (NIST14) mass spectral libraries (percentage match of 70% was set as the accepted mass error). Pathway analysis was performed using MetaboAnalyst 3.0 18 (http://www.MetaboAnalyst. ca/).

### 4.12. UPLC–QTOF-MS-Based Untargeted Metabolomics Analysis for Quail Samples

#### 4.12.1. Quail Sample Handling

Firstly, 200 μL of quail samples and 600 μL of methanol were mixed and kept at 4 °C before use. The samples were centrifuged at 12,000 rpm for 10 min at 4 °C, and the supernatant was collected. Then, samples were filtered through a syringe filter (0.22 μm) and injected into the UPLC–QTOF-MS system.

#### 4.12.2. Chromatography and Mass Spectrometry Conditions

The chromatographic analysis was performed using an Agilent 6550 iFunnel Q-TOF LC–MS (Agilent Technologies, CA, USA). A 4-μL aliquot of each sample was injected onto a ZORBOX RRHD C18 analytical column (2.1 mm inner diameter (i.d.) × 100 mm, 1.7 μm i.d., Agilent Technologies, USA), with a column temperature of 30 °C. For the ESI+ analysis, separation was obtained with a 25-min linear gradient with mobile phases of solvent A (acetonitrile spiked with 0.1% formic acid) and solvent B (water spiked with 0.1% formic acid). The flow rate was set as 0.30 mL/min. The gradient was set as follows: a linear gradient of 5% A over initial 1.0 min, 5–40% A over 1.0–9.0 min, 40–90% A over 9.0–19.0 min, 90–100% A over 19.0–21.0 min, and 100% A over 21.0–25.0 min. The eluent was introduced to the mass spectrometer directly. Each sample was pooled as a quality control (QC) and injected every 10 samples.

The Agilent 6550 Q-TOF/MS with an electrospray ionization source (ESI) was used for mass spectrometry. Electrospray was used for ionization. The parameters in positive ionization mode were as follows: the electrospray capillary voltage was 4.0 kV; the mass range was set from 80 to 1000 *m*/*z*; The gas temperature was 225 °C; the gas flow was 11 L/min; The nebulizer was set to 45 psig; The sheath gas temperature was 350 °C, and the sheath gas flow was 12 L/min; The nozzle voltage was 500 V. Reference masses 121.0509 (Purine, (C_5_H_4_N_4_ + H)^+^) and 922.0098 (HP-0921, (C_18_H_18_O_6_N_3_P_3_F_24_ + H)^+^) were used for internal mass calibration during the MS analysis.

#### 4.12.3. Data Processing and Pattern Recognition Analysis

Profinder (version B.06.00, Agilent Technologies, Santa Clara, CA, USA) was used for data processing. Up to 2000 compounds were extracted. Data filtering, data normalization, missing value estimation, and fold changes were obtained using the MetaboAlalyst 3.0 online software. Resultant data matrices were analyzed by the SIMCA-P+ 13.0 (Umetrics, Umeå, Sweden) software. The significant differences were screened using the fold change value (>1.5) combined with the *t*-test (*p* < 0.05) and ANOVA (*p* < 0.05). Variables with significant changes were determined as potential biomarkers for further identification of molecular formulas. Biomarkers were tentatively identified by the online METLIN database. Pathway analysis was performed using MetaboAnalyst 3.0 18 (http://www.MetaboAnalyst. ca/).

### 4.13. PLA2, Glycerol, NEFA, GSH, GSH-Px, GR, γ-GCS, γ-GT, Total AOC, MDA, and SOD Assays

Samples were obtained by ultrasonication for 1 min at 4 °C. The phospholipase A2 (PLA2), glycerol, non-esterified free fatty acid (NEFA), GSH, GSH-PX, GR, γ-GCS, γ-GT, total AOC, MDA, and SOD levels were measured according to guides in respective kits (Nanjing Jiancheng, Nanjing, China; CAS numbers H243, F005-2-1, A042-2-1, A006-2, A005, A062, A091-1, C017-2, A015-3, A003-3, and A001-3) using a BIORAD 550 spectrophotometer plate reader.

### 4.14. Reactive Oxygen Species (ROS) Assay

Firstly, 2′,7′-dichlorodihydrofluorescein diacetate (DCFH-DA) was applied to measure intracellular reactive oxygen species (ROS). The suspension was collected after centrifugation (9000× *g*, 5 min) and washed with PBS. Cells were added to DCFH-DA for 30 min in the dark. The fluorescence intensity of dichlorofluorescein (DCF), was measured with an excitation wavelength of 488 nm and an emission wavelength of 510 nm using a fluorescence spectrophotometer (ECLIPSE Ts2R-FL, Nikon, Japan).

### 4.15. DAPI Staining

T24 or HUVEC cells were seeded in 24-well plates and washed with PBS, before being fixed with 4% paraformaldehyde. Then, cells were dyed by 4’,6-diamidino-2-phenylindole (DAPI) for 5 min in the dark. Cells were observed with an excitation wavelength of 358 nm and an emission wavelength of 461 nm using a fluorescence spectrophotometer (ECLIPSE Ts2R-FL, Nikon, Japan).

### 4.16. Statistical Analysis

The SPSS software program (version 22.0, Chicago, IL, USA) was applied for data analysis. The evaluation of significant differences in the results was done using Student’s *t*-test and one-way analysis of variance (ANOVA) with a post hoc test (when the *t*-test was not suitable, the Mann–Whitney U test was applied). The differences were deemed as statistically significant and highly significant when *p* < 0.05 and *p* < 0.01, respectively. Because the metabolites with small *p*-values were tested by building the classification model, false discovery rate (FDR) correction was not applied during the univariate metabolomics analysis.

## Figures and Tables

**Table 1:** Effects of a ligustrazine–betulinic acid derivative (BA-12) on apoptosis and cell cycle of human bladder carcinoma (T24) cells. G—gap phase; M—mitosis phase; S—synthesis phase.

Group	Apoptosis Ratio (%)	G0/G1 (%)	S (%)	G2/M (%)
Control	5.7 ± 1.2	62.5 ± 6.7	9.4 ± 1.8	27.6 ± 3.4
Dissolvent	7.2 ± 1.5	62.1 ± 5.1	9.2 ± 1.3	29.0 ± 3.8
Dovitinib (2.5 μM)	25.5 ± 5.1 **	50.8 ± 4.2 *	11.1 ± 1.5	37.5 ± 4.4 *
BA-12 (2.5 μM)	20.3 ± 4.2 *	51.7 ± 4.4 *	10.3 ± 1.6	38.7 ± 4.0 *

ANOVA with a post hoc test was used to calculate the significance of the differences; * *p* < 0.05, ** *p* < 0.01 compared with the dissolvent group. Experiments were executed three times. Results are displayed as means ± SD.

**Table 2:** Differential identified metabolites for discrimination among control and BA-12 groups based on GC–MS metabolomics. RT—retention time; VIP—variable importance for projection; MEOX—methoxime; TMS—trimethylsilyl.

RT (min)	Actual *m*/*z*	Metabolite	*m*/*z*	Formula	Derivative Type	Derivative Weight/Formula	Predicted *m*/*z*	VIP	*p* (corr)
3.38	236.5340	Chalcone (HMDB0003066)	208.2552	C_15_H_12_O	1 MEOX	C_16_H_15_NO	237.1154	1.38	0.90
5.53	234.4430	Melatonin (HMDB0001389)	232.2783	C_13_H_16_N_2_O_2_	Underivatized			1.16	0.65
16.70	340.5060	5-Methylcytosine (HMDB0002894)	125.1286	C_5_H_7_N_3_O	3 TMS	C_14_H_31_N_3_OSi_3_	341.6720	9.19	0.98
19.80	86.1770	Putrescine (HMDB0001414)	88.1515	C_4_H_12_N_2_	Underivatized			4.42	−0.52

The significant differences were generated from a Student’s *t*-test or Mann–Whitney U test when the Student’s *t*-test was not suitable.

**Table 3:** Altered metabolic pathways regulated by BA-12 based on GC–MS metabolomics.

Pathway	Raw *p*	Total	Hits
Glutathione metabolism	0.0617	38	1
Pyrimidine metabolism	0.0961	60	1
Arginine and proline metabolism	0.1220	77	1
Tryptophan metabolism	0.1250	79	1

The significant differences were generated from a Student’s *t*-test or Mann–Whitney U test when the Student’s *t*-test was not suitable.

**Table 4:** Differential identified metabolites for discrimination among control and BA-12 groups based on UPLC–TOF-MS metabolomics. KEGG ID—Kyoto Encyclopedia of Genes and Genomes identifier; PC—phosphatidylcholine.

RT (min)	*m*/*z*	Metabolite	KEGG ID	Formula	VIP	*p* (corr)
3.10	154.0373	Octadecanamide	C13846	C_4_H_12_N_2_	3.76	−0.83
15.32	496.3440	8-Azaxanthine	C04598	C_15_H_12_O	3.84	−0.90
17.18	524.3722	PC (0:0/16:0) (HMDB0010382)	C04230	C_24_H_50_NO_7_P	2.31	−0.89
20.07	284.2902	LysoPC (18:0)	C04230	C_26_H_54_NO_7_P	3.21	−0.73

The significant differences were generated from a Student’s *t*-test or Mann–Whitney U test when the Student’s *t*-test was not suitable.

**Table 5:** Altered metabolic pathways regulated by BA-12 based on UPLC–TOF-MS metabolomics. FDR—false discovery rate.

Pathway	Raw *p*	FDR	Impact
Glycerophospholipid metabolism	0.000143	0.011	0.27873

The significant differences were generated from a Student’s *t*-test or Mann–Whitney U test when the Student’s *t*-test was not suitable.

**Table 6:** The mass data of glutathione (GSH) detected in quail samples by UPLC–QTOF-MS.

Index	*m*/*z*	Chemical Formula	Predicted *m*/*z* (M + H)^+^	Actual *m*/*z*	RT (min)	Integral Area (Mean ± SD)	
						Control Group	BA-12 (80 μg) Group
GSH	307.3230	C_10_H_17_N_3_O_6_S	308.1000	308.2884	19.10	58,713 ± 5928	33,773 ± 3356
Glycerophospholipid	170.0578	C_3_H_7_O_6_P	171.0578	171.0649	0.78	15,994 ± 1191	12,452 ± 1332

## Supplementary Materials

The following are available online at https://www.mdpi.com/1422-0067/20/16/4062/s1.

## Abbreviations

TCM	traditional Chinese medicine
BA-12	ligustrazine–betulinic acid derivative
GSH	glutathione
qCAM	quail chick chorioallantoic membrane
BSTFA	*N*,*O*-bis(trimethylsilyl)trifluoroacetamide
TMCS	trimethylchlorosilane
DAPI	4’,6-diamidino-2-phenylindole
GR	glutathione reductase
total AOC	total antioxidant capacity
γGT	γ-glutamyl transferase
γGCS	γ-glutamylcysteine synthetase
