# Correction: Cui, H., et al. BA-12 Inhibits Angiogenesis via Glutathione Metabolism Activation. *Int. J. Mol. Sci.* 2019, *20*, 4062

**DOI:** 10.3390/ijms21186814

**Published:** 2020-09-17

**Authors:** Herong Cui, Wenbo Guo, Beibei Zhang, Guoping Li, Tong Li, Yanyan Yuan, Na Zhang, Yuwei Yang, Wuwen Feng, Fuhao Chu, Shenglan Wang, Bing Xu, Penglong Wang, Haimin Lei

**Affiliations:** 1 School of Chinese Pharmacy, Beijing University of Chinese Medicine, Beijing 102488, China; 2 School of Pharmacy, Chengdu University of Traditional Chinese Medicine, Chengdu 610000, China; 3 School of Acupuncture and Massage, Beijing University of Chinese Medicine, Beijing 102488, China

The authors wish to make the following corrections to this paper [1]:

In Figure 3B, the flow cytometry chart of Dovitinib is the same as that of BA-12 2.5 μM. This was an error made during figure construction. The corrected Figure 3 is shown below (Figure 1).

The authors would like to apologize for any inconvenience caused to the readers by these changes. These changes have no material impact on the conclusions of our paper. We apologize to our readers.

## Figures and Tables

**Figure 1:**
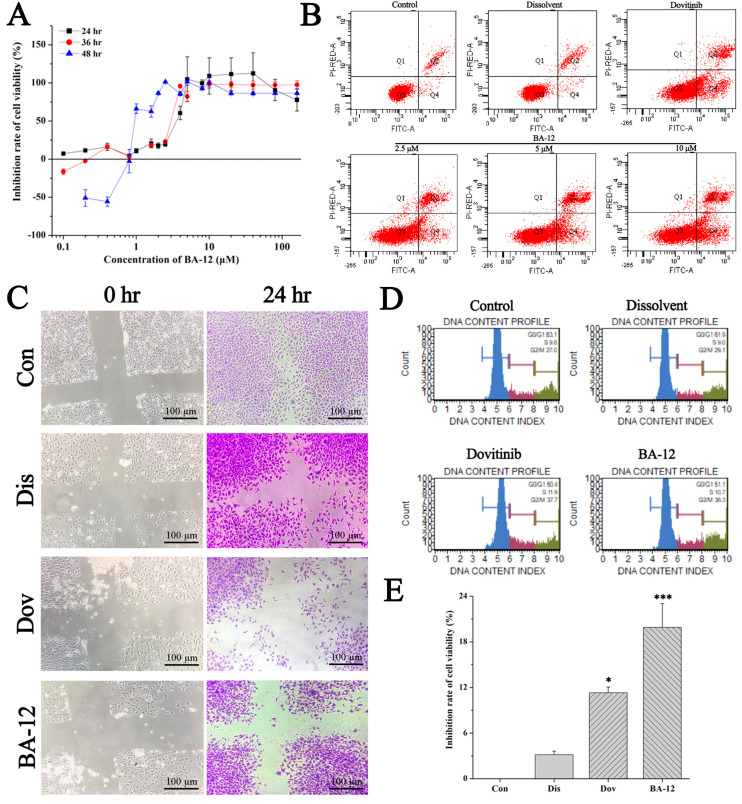
The in vitro antitumor activity of BA-12 on T24 cells. (**A**) Inhibition rate of cell viability of T24 cells for MTT assays (cells treated with BA-12 at doses of 0.25–160 μM) for 24, 48, and 72 h. (**B**) Apoptosis analysis of T24 cells induced by agents using Annexin V-fluorescein isothiocyanate (FITC)/propidium iodide (PI) staining. (**C**) Results for wound scratch assay (cells treated with BA-12 at doses of 2.5 μM) after 24 h under the microscope (100×). The most representative fields are shown. (**D**) Cell cycle analysis using PI staining (cells treated with BA-12 at doses of 2.5 μM). (**E**) Inhibition rate of cell viability of T24 cells for MTT assays (cells treated with BA-12 at doses of 2.5 μM) for 24 h. ANOVA with a post hoc test was used to calculate the significance of the differences; * *p* < 0.05, *** *p* < 0.001 compared with the dissolvent group. Experiments were executed three times. Results are displayed as means ± SD.
