# Supplementary material for: BA-12 Inhibits Angiogenesis via Glutathione Metabolism Activation
Source: Int J Mol Sci. 2019 Aug 20;20(16):4062. doi: 10.3390/ijms20164062 (PMC6720627; doi:10.3390/ijms20164062)
Supplement: Supplementary file 1 [file ijms-20-04062-s001.zip › ijms-563296-supplementary.pdf]

BA-12 Inhibits Angiogenesis via Glutathione Metabolism Activation

Herong Cui [1], Wenbo Guo [1], Beibei Zhang [1], Guoping Li [1], Tong Li [1], Yanyan Yuan [1], Na Zhang [1], Yuwei Yang [1], Wuwen Feng [2], Fuhao Chu [1], Shenglan Wang [3], Bing Xu [1,*], Penglong Wang [1,*] and Haimin Lei [1,*]

[1] School of Chinese Pharmacy, Beijing University of Chinese Medicine, Beijing 102488, China
[2] School of Pharmacy, Chengdu University of Traditional Chinese Medicine, Chengdu 610000, China
[3] School of Acupuncture and Massage, Beijing University of Chinese Medicine, Beijing 102488, China
* Corresponding: weichenxubing@126.com (B.X.); wpl581@126.com (P.W.); hm_lei@126.com (H.L.); Tel.: +86-10-8473-8645 (H.L.); Fax: +86-10-8473-8645 (H.L.)

A list of figures and tables in the Supplementary Information

No.	Headline	Figures/Tables
**1**	The chemical information of BA-12 by UPLC-MS	Figure S1;
**2**	Desorption electrospray ionization mass spectrometry (DESI-MS) imaging of BA-12 in qCAM samples	Figure S2; Table S1
**3**	Vessel number and vessel area analysis for qCAM samples	Figure S3
**4**	Analysis for wound scratch and tube formation assay	Figure S4
**5**	The total ion chromatograms (TIC) of intracellular fingerprint	Figure S5
**6**	The parameters of PCA and OPLS-DA model	Table S2 Figure S6
**7**	The score plots of PCA analysis contained QC samples	Figure S7
**8**	UPLC-QTOF-MS based metabolomics analysis	Figure S8 Table S3
**9**	Macroscopical observation of Caenorhabditis elegans 240 h after PBS or dissolvent or dovitinib or BA-12 treatment in the experiment	Figure S9
**10**	The compliance of the grouping of gels/blots cropped from different parts of the same gel, or from different gels, fields in the main manuscript	Figure S10
**11**	The effect of BA-12 on REDOX balance	Figure S11
**12**	Prediction pathways regulated by BA-12	Table S4

1. The chemical information of BA-12 by UPLC-MS.

The chromatographic analysis of BA-12 was performed on an Agilent 1290 series UHPLC system coupled to 6550 Q-TOF/MS mass spectrometer. The analysis was conducted on a ZORBAX RRHD 300 SB-C18 column (2.1×100 mm, 1.8 µm). For the ESI+ analysis, the mobile phases used were solvent A (Acetonitrile spiked with 0.1% formic acid), solvent B (Water spiked with 0.1% formic acid), with gradient elution as follows: 25% A at 0 – 15 min, 25% – 60% A at 15 – 20 min, 60 % – 95% A at 20–25 min, 95% A at 25–30 min. The flow rate was kept at 0.3 mL/min. The column and autosampler were maintained at 30ºC and 4°C, respectively. The injection volume of reference compounds and samples was 1 µL. The eluent was introduced to the mass spectrometer directly. The mass range was set from m/z 360 to 700. The optimal conditions of analysis were as follows: the capillary voltage was 4 kV; desolvation gas flow was 11.0 L/min, the source temperature was set at 125ºC, the desolvation gas temperature was 225ºC. Calibrations were automatically conducted from m/z 360 to 700 with a solution of sodium formate. The chemical information and a base peak chromatogram are shown as Supplementary **Figure S1**.

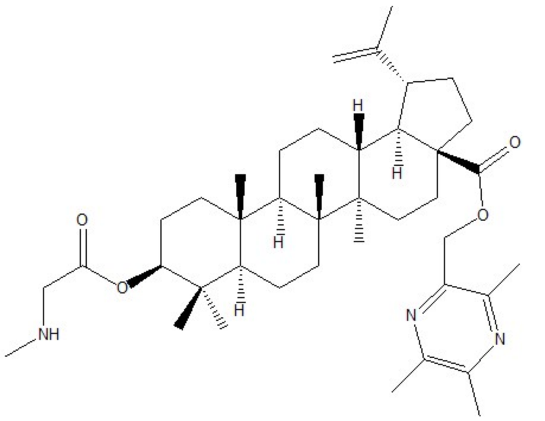

$C_{41}H_{63}N_3O_4$
Exact Mass: 661.48
Mol. Wt.: 661.96
m/e: 661.48 (100.0%), 662.49 (46.7%), 663.49 (11.5%), 664.49 (2.1%), 662.48 (1.1%)
C, 74.39; H, 9.59; N, 6.35; O, 9.67

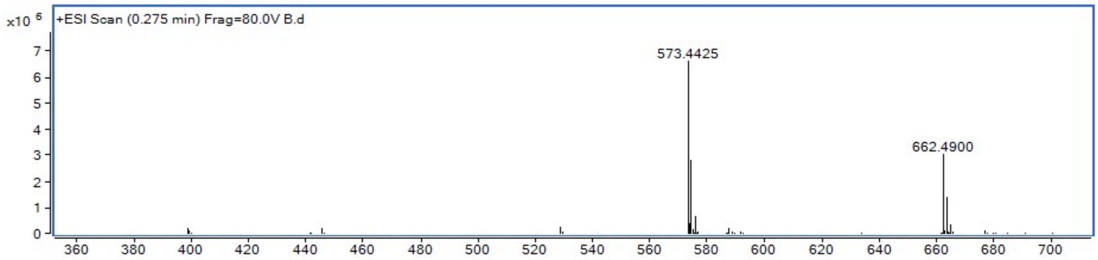

Figure S1 | The chemical information and a base peak chromatogram of the UPLC-MS analysis of BA-12 in the ESI+ mode.

2. **Desorption electrospray ionization mass spectrometry (DESI-MS) imaging of BA-12 in qCAM samples.**

Desorption electrospray ionization mass spectrometry (DESI-MS) imaging was used for quality control by detecting BA-12 in the quail chick chorioallantoic membrane (qCAM) sections. The mass range was set from m/z 100 to 1000. The results are shown as Supplementary **Figure S2** and **Table S1**.

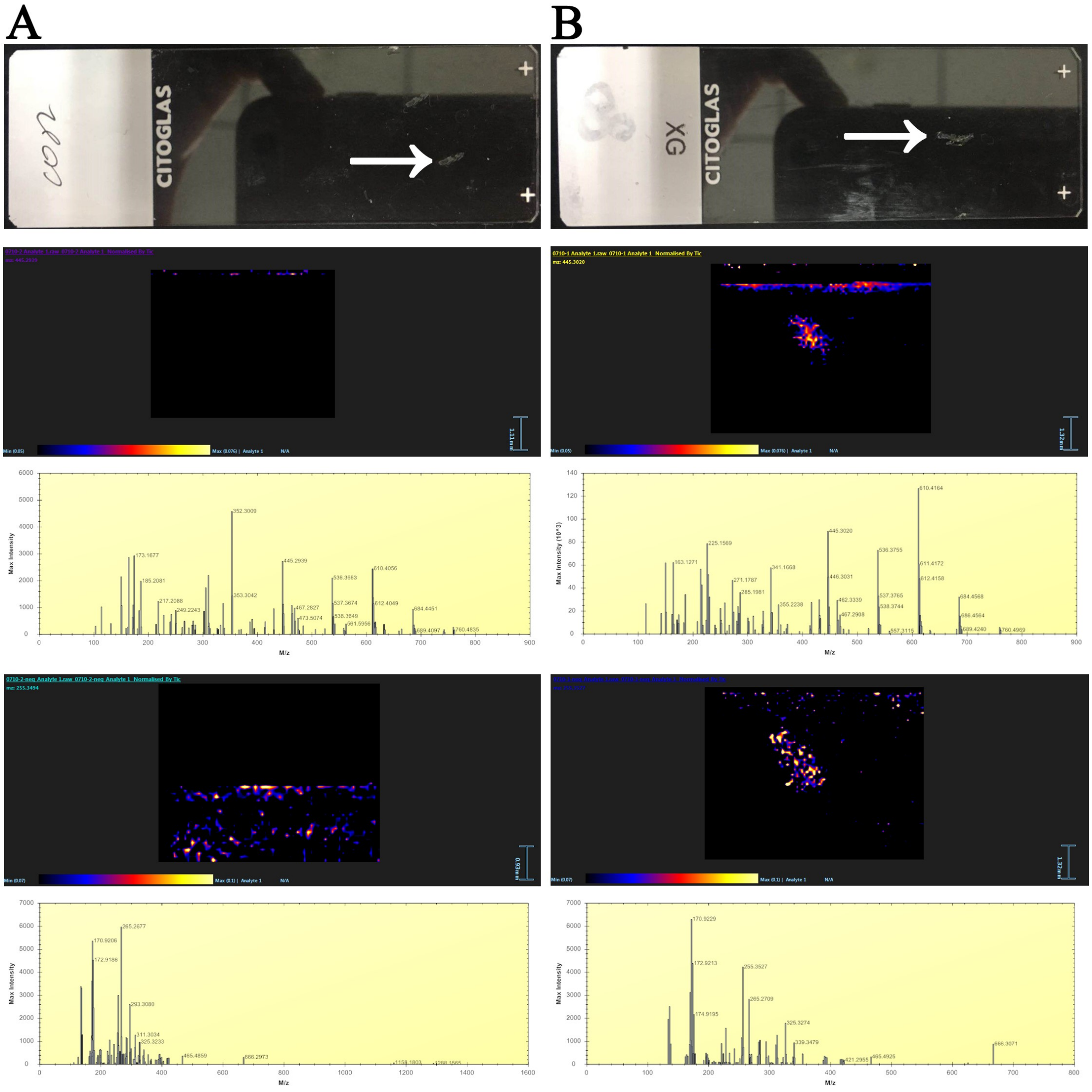

Figure S2 | Imaging mass spectrometry of qCAM samples of control (A) and BA-12 (80 μg) (B) group.

Table S1 | The mass data of imaging mass spectrometry

Control group				BA-12 (80 μg) group			
Positive mode		Negtive mode		Positive mode		Negtive mode	
M/z	Max Intensity	M/z	Max Intensity	M/z	Max Intensity	M/z	Max Intensity

610.4056	2446	170.9206	5362	610.4164	126790	170.9229	6308
611.4067	1365	265.2677	5974	536.3755	72889	172.9213	4385
536.3663	2100	172.9186	4533	611.4172	61040	265.2709	2832
185.2081	1984	168.9228	3623	445.302	89520	134.9414	2517
612.4049	1143	134.9395	3320	612.4158	48146	168.925	3130
309.2641	2198	255.3494	2995	684.4568	32270	132.943	1961
173.1677	2930	132.9413	3375	537.3765	33126	255.3527	4231
684.4451	943	174.9171	2457	163.1271	62199	174.9195	2168
537.3674	1157	293.308	2600	149.106	62063	169.9235	1048
445.2939	2727	253.3334	1369	446.3031	49586	293.3117	989
182.2825	890	311.3034	1275	185.2115	34471	253.3362	1153
217.2088	1231	281.3736	1167	685.4578	10245	281.3771	972
227.2328	722	169.9208	1251	462.3339	29349	311.3073	1269
685.4458	337	325.3233	971	538.3744	23675	325.3274	1794
163.1245	2861	136.9377	1294	429.2658	29774	136.9396	889
538.3649	669	171.9196	1051	309.2699	15547	171.9222	768
171.1875	1098	283.3897	1141	686.4564	15737	167.9252	874
149.1036	2149	167.923	1147	613.4158	16327	283.3932	1053
613.4053	419	339.3436	635	447.3012	23719	339.3479	944
249.2243	892	227.3088	1065	173.1706	16688	309.3119	849
352.3009	4579	309.3079	749	467.2908	16631	227.3118	1568
686.4445	355	241.3293	875	182.286	9884	334.1297	69

301.2719	870	391.2506	444	341.1668	57748	216.206	81
353.3042	1439	389.2505	424	113.1276	26447	391.2572	322
265.2243	495	266.2713	1197	249.2286	22326	389.257	353
241.2167	755	297.2833	686	225.1569	78818	241.3323	682
412.4858	260	176.9157	599	615.3806	10993	176.9178	470
281.2969	502	256.3535	818	463.3347	12057	297.287	376
473.5074	596	173.9185	592	217.2126	7700	173.9207	485
467.2827	981	269.3698	448	301.277	10870	266.2744	441
446.2949	1129	417.285	267	281.1803	24013	208.3948	64
557.3017	228	194.9462	625	227.2369	12571	162.9274	425
631.3409	378	353.3483	429	150.118	19053	256.3561	751
462.3256	1074	393.2511	281	412.493	7228	417.2912	225
615.3696	468	419.2862	277	430.2666	16350	419.2932	213
113.1259	1027	279.3573	470	687.4561	6979	269.3726	439
413.4311	473	162.925	586	539.3749	8339	393.2578	297
447.4679	317	337.3477	384	213.1183	56594	205.6349	89
250.2321	408	312.307	326	265.2281	7725	233.2671	493
325.2438	223	294.3116	541	250.2361	11985	279.3607	367
389.4087	563	196.9442	660	355.2238	25414	234.45	72
413.4878	245	210.3095	65	614.4144	7317	465.4925	329
429.2578	958	254.3369	491	171.1905	9682	353.3527	460
539.3674	370	326.3269	348	464.3327	12233	164.9258	363

365.2893	169	465.4859	364	631.3516	3195	327.9077	76
130.232	407	321.3485	394	541.3354	7902	190.95	175
447.2931	775	415.28	259	758.4973	5839	326.1312	77
740.5228	111	233.2636	407	281.302	8286	195.9098	318
614.4041	235	324.2624	75	170.147	12419	267.3569	298
213.2486	376	164.9237	493	413.4378	4489	225.6266	86
687.4443	224	223.134	394	431.2641	13535	337.3519	329
541.3251	395	267.3534	415	365.2863	2028	215.5975	85
170.1441	646	282.3772	331	227.1531	51593	197.9077	283
758.4844	267	272.9509	459	241.2206	4850	312.3108	286
310.2687	428	267.2668	417	161.147	17073	247.33	83
338.4841	232	343.3551	115	141.1706	18005	223.1365	472
285.2571	352	171.2266	510	299.2242	13945	207.7618	141
522.7936	214	387.2424	222	557.3115	2481	326.3308	444
331.3489	345	197.9046	401	223.1735	12004	300.1463	67
483.2608	322	666.2973	307	271.1787	46753	302.2287	87
312.4971	219	206.9985	69	448.3016	7720	282.3808	446
632.3418	174	284.3936	346	325.249	8244	294.3151	311
505.5239	183	198.9431	623	215.1142	42753	321.3525	298
261.2491	257	421.2879	277	353.3098	3532	197.588	69
393.4561	226	188.9505	405	415.2093	27414	415.2848	215
279.216	341	195.907	309	616.3778	6876	210.8195	90

633.3401	167	160.9259	355	468.2921	6706	224.5121	84
741.5244	174	109.5707	76	483.2699	3683	666.3071	884
281.1786	350	397.3877	310	759.4984	4632	232.3251	92
429.4104	178	307.1353	219	285.1981	36197	254.34	393
558.3024	149	305.1381	230	279.2208	11330	304.3123	61
616.3668	273	187.0079	295	283.1671	18185	218.0213	85
336.2652	1155	377.235	194	207.1323	10818	318.1434	81
102.1893	306	220.6196	66	338.4904	1589	250.2623	255
239.2714	475	363.2143	232	327.2284	11885	320.2245	83
759.4851	160	340.3472	255	429.4202	5041	217.2436	92
171.2363	230	381.3889	188	689.424	3915	244.1237	81
284.4569	177	123.9721	314	413.4942	3524	194.9489	492
161.1445	270	189.0053	344	519.3432	4776	227.9016	105
689.4097	131	223.9037	195	312.5024	3648	160.9281	337
186.2123	281	220.2519	579	371.2437	8524	209.3216	89
273.2895	244	390.2526	172	327.1109	19947	195.0259	329
179.1568	209	205.6616	55	359.1725	7987	337.4595	134
475.5014	151	260.0682	73	164.1334	8711	624.6671	63
309.3382	293	222.5853	68	251.1645	9369	196.9462	433
559.3007	134	250.26	298	473.5136	4367	231.8253	79
468.2835	502	200.9053	70	273.1761	19148	227.1251	95
463.3265	781	392.2542	267	369.2951	4670	421.2955	186

304.4314	1741	1288.1565	42	760.4969	3206	215.9992	102
369.2833	186	403.2722	144	239.2752	7818	220.5539	96
658.7547	132	245.2575	68	327.3969	5832	420.2969	123
327.2188	194	316.3174	86	343.1608	18820	199.9053	184
663.6987	203	361.2165	167	173.2567	9033	220.2541	909
150.1141	1073	279.0954	254	261.2503	2715	387.248	179
385.4476	471	309.1346	213	632.3525	2233	245.2638	79
742.5231	83	183.638	80	679.7484	4268	267.2699	273
760.4835	167	1158.1803	69	229.153	32335	340.3514	290
561.5956	389	170.9733	353	342.1678	18388	269.8024	82
474.5081	182	327.3225	190	257.1572	27175	228.673	85
158.1801	227	260.9917	567	393.4634	2207	204.6894	82

3. Vessel area and vessel number analysis for qCAM samples.

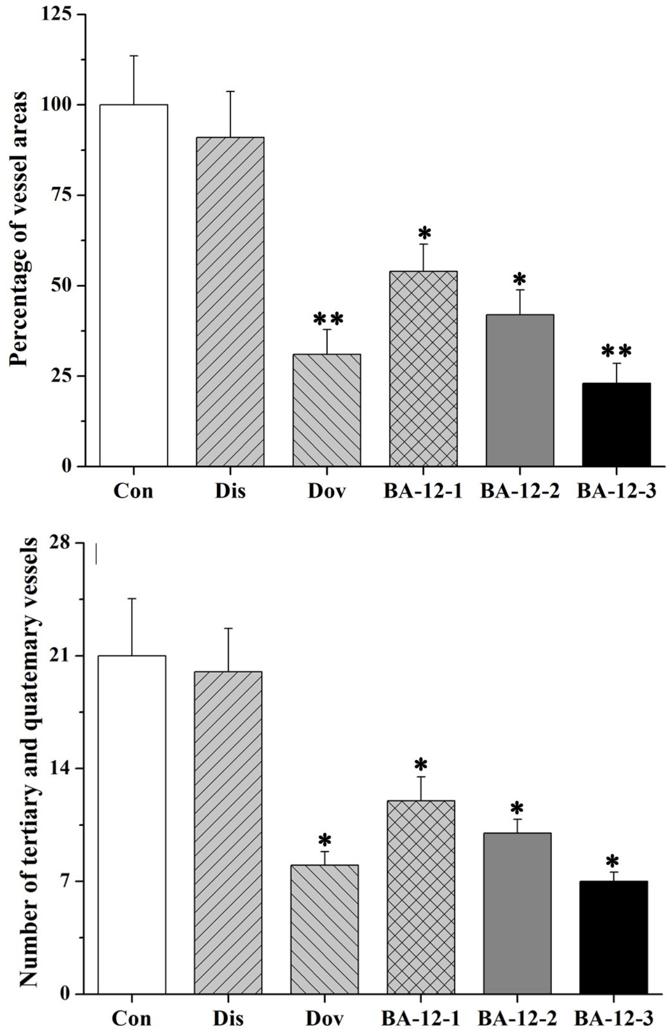

Figure S3 | Vessel area and vessel number analysis for qCAM samples. Con, control group treated with PBS; Dis, dissolvent group treated with dissolvent contained 0.5 % DMSO; Pos, positive control group treated with dovitinib (40 μg); BA-12 1-3, groups treated with BA-12 at doses of 20, 40, 80 μg, respectively. ANOVA with the post hoc test was used to calculate the significance of the differences, *, ** and *** represents $p < 0.05$, $p < 0.01$, and $p < 0.001$ compared with the dissolvent group, respectively. All experiments were performed 3 times, and the results are expressed as the mean ± S.D.

4. Analysis for wound scratch and tube formation assay.

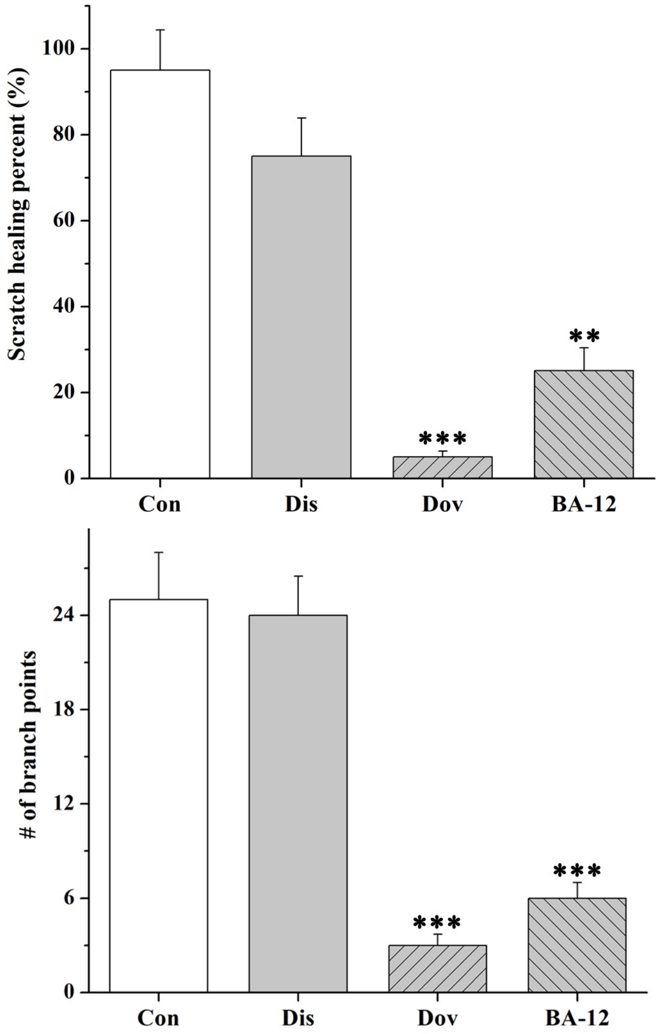

Figure S4 | Analysis for wound scratch and tube formation assay by HUVEC. Con, control group treated with PBS; Dis, dissolvent group treated with dissolvent contained 0.5 % DMSO; Pos, positive control group treated with dovitinib (2.5 μM); BA-12 groups treated with BA-12 at doses of 2.5 μM. ANOVA with the post hoc test was used to calculate the significance of the differences, *, ** and *** represents $p < 0.05$, $p < 0.01$, and $p < 0.001$ compared with the dissolvent group, respectively. All experiments were performed 3 times, and the results are expressed as the mean ± S.D.

5. The total ion chromatograms (TIC) of intracellular fingerprint.

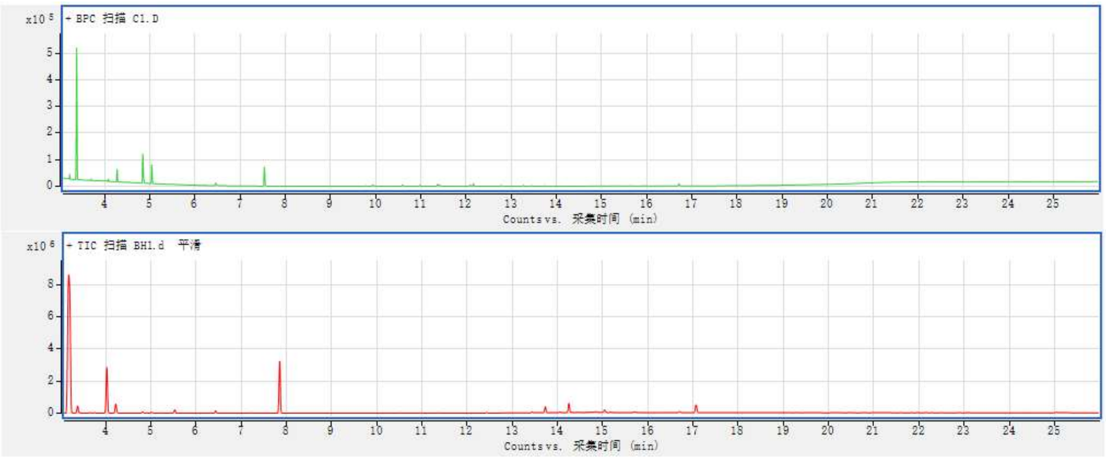

Figure S5 | The total ion chromatograms (TIC) of intracellular fingerprint (First: Control group; Second: BA-12 group).

6. The parameters of PCA and OPLS-DA model.

Table S2 | The parameters of PCA and OPLS-DA model

Model	Component	R2X	R2Y	Q2Y	R2-intercept	Q2-intercept
PCA (M1)	2	0.465	—	-0.204	—	—
PLS-DA (M2)	1+1+0	0.400	0.993	0.873	0.043	0.5

A

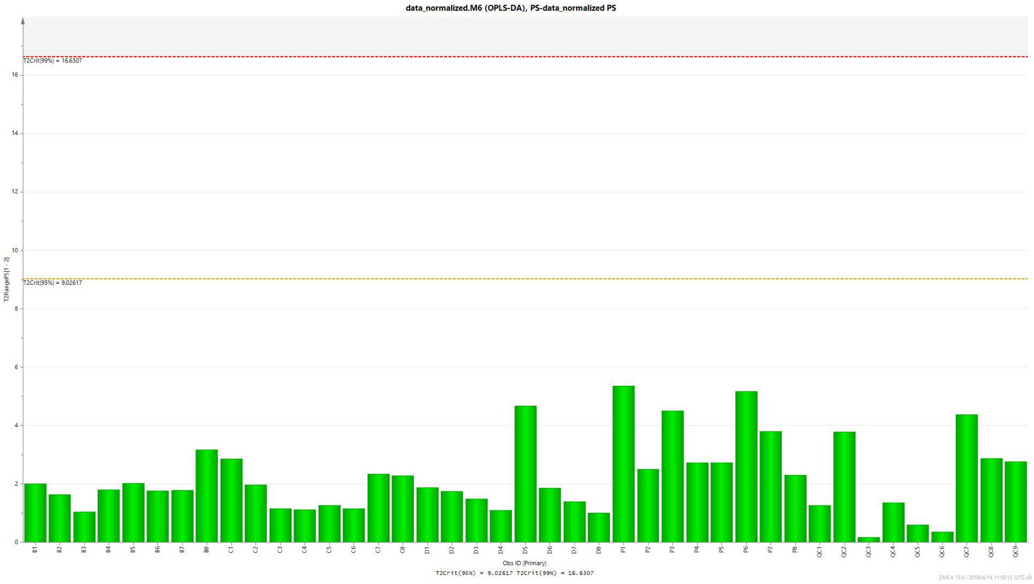

B

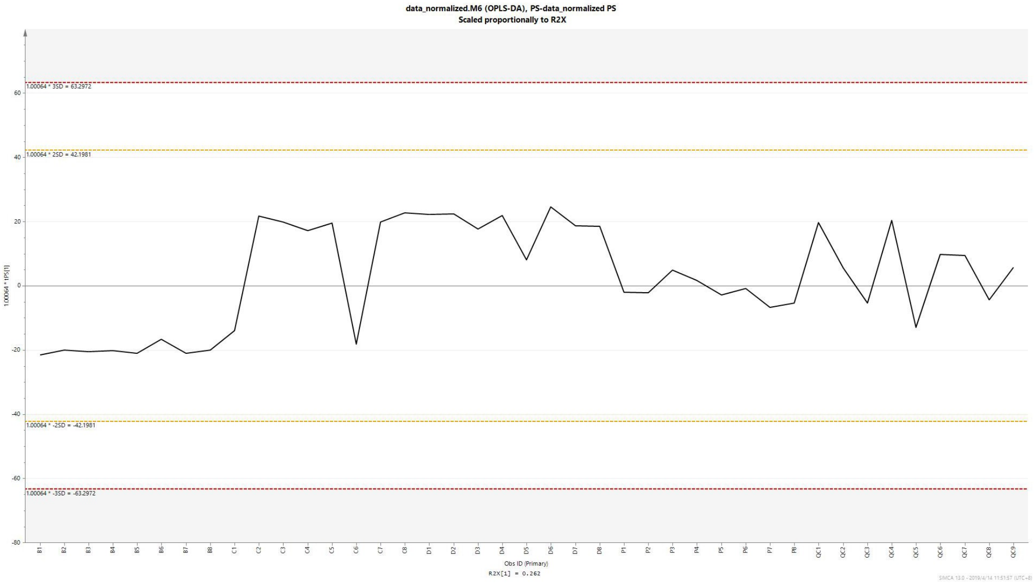

Figure S6 | Hotelling's T2 (A) and score plot (B) of OPLS-DA(M2).

7. The score plots of PCA analysis contained QC samples.

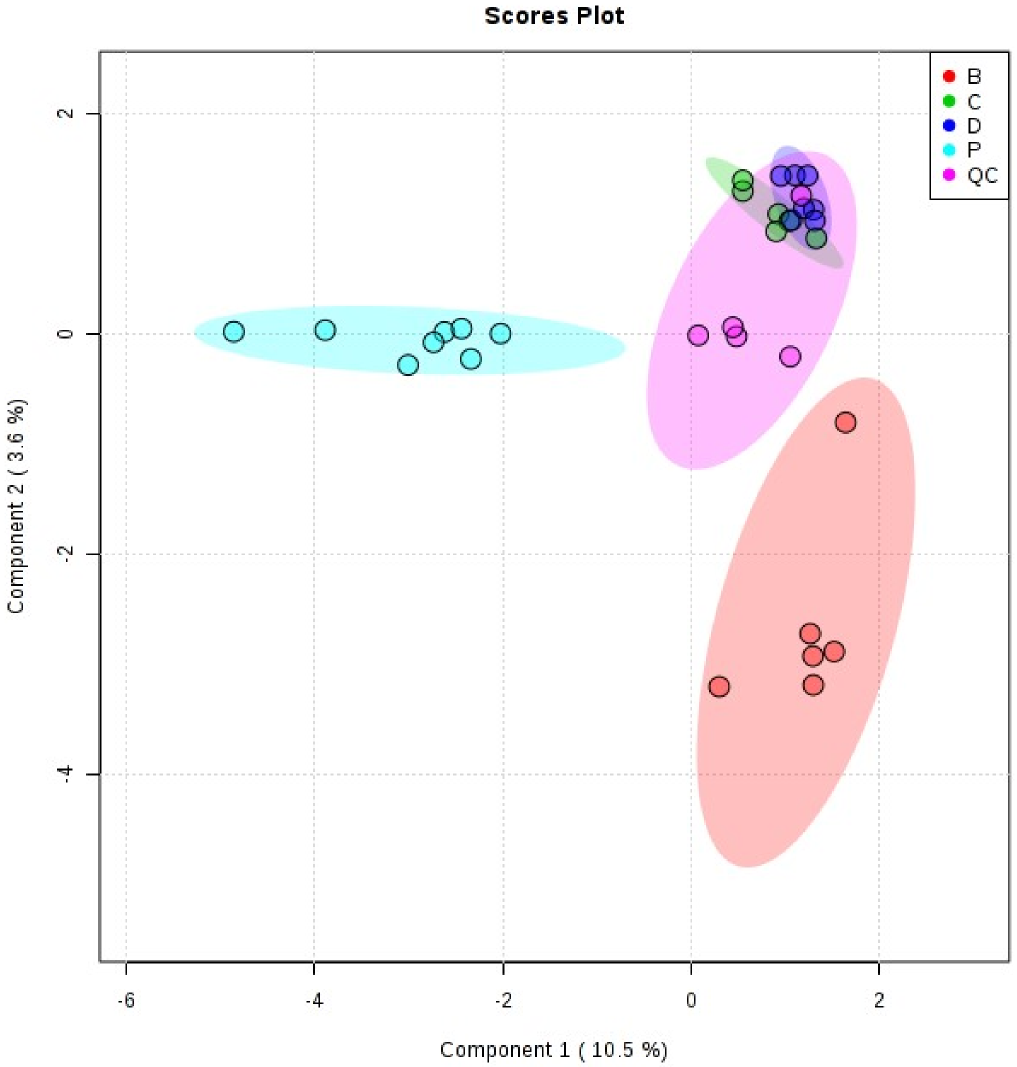

Figure S7 | The score plots of PCA analysis contained QC samples.

8. UPLC-QTOF-MS based metabolomics analysis.

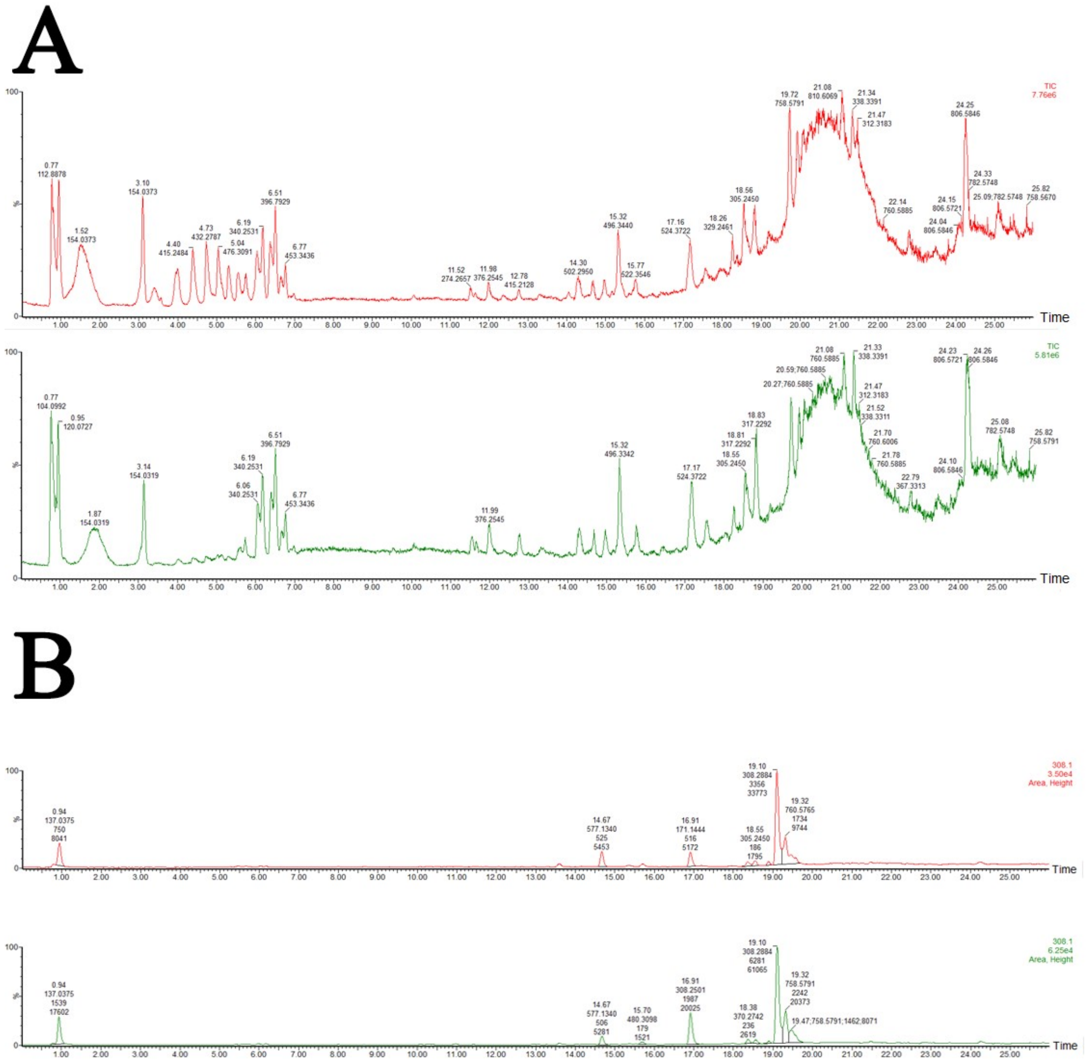

Figure S8 | The total ion chromatograms (TIC) of intracellular fingerprint (First: Control group; Second: BA-12 group). (**A**) The total ion chromatograms (TIC) of intracellular fingerprint (First: Control group; Second: BA-12 group). (**B**) Intracellular chromatograms extracted from TIC for GSH (308.1000) in (First: Control group; Second: BA-12 group).

Table S3 | The parameters of PCA and OPLS-DA model

Model	Component	R2X	R2Y	Q2Y	R2-intercept	Q2-intercept
PCA (M1)	2	0.611	—	0.167	—	—
PLS-DA (M2)	1+1+0	0.954	0.999	0.992	0.001	0.5

9. **Macroscopical observation of Caenorhabditis elegans 240 h after PBS or dissolvent or dovitinib or BA-12 treatment in the experiment.**

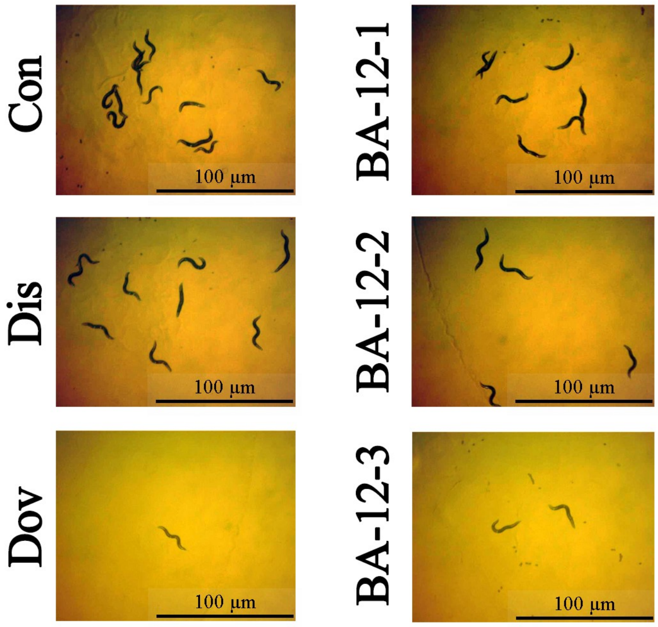

Figure S9 | Macroscopical observation of Caenorhabditis elegans 240 h after PBS or dissolvent or dovitinib or BA-12 treatment in the experiment as indicated. Con, control group treated with PBS; Dis, dissolvent group treated with dissolvent contained 0.5 % DMSO; Pos, positive control group treated with dovitinib (40 μg); BA-12 1-3, groups treated with BA-12 at doses of 20, 40, 80 μg, respectively.

10. **The compliance of the grouping of gels/blots cropped from different parts of the same gel, or from different gels, fields.**

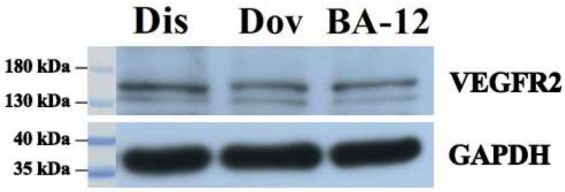

Figure S10 | The compliance of the grouping of gels/blots cropped from different parts of the same gel, or from different gels, fields. The compliance of the grouping of gels/blots for VEGFR2 and GAPDH 36 h after dissolvent or dovitinib or BA-12 treatment.

11. The effect of BA-12 on REDOX balance.

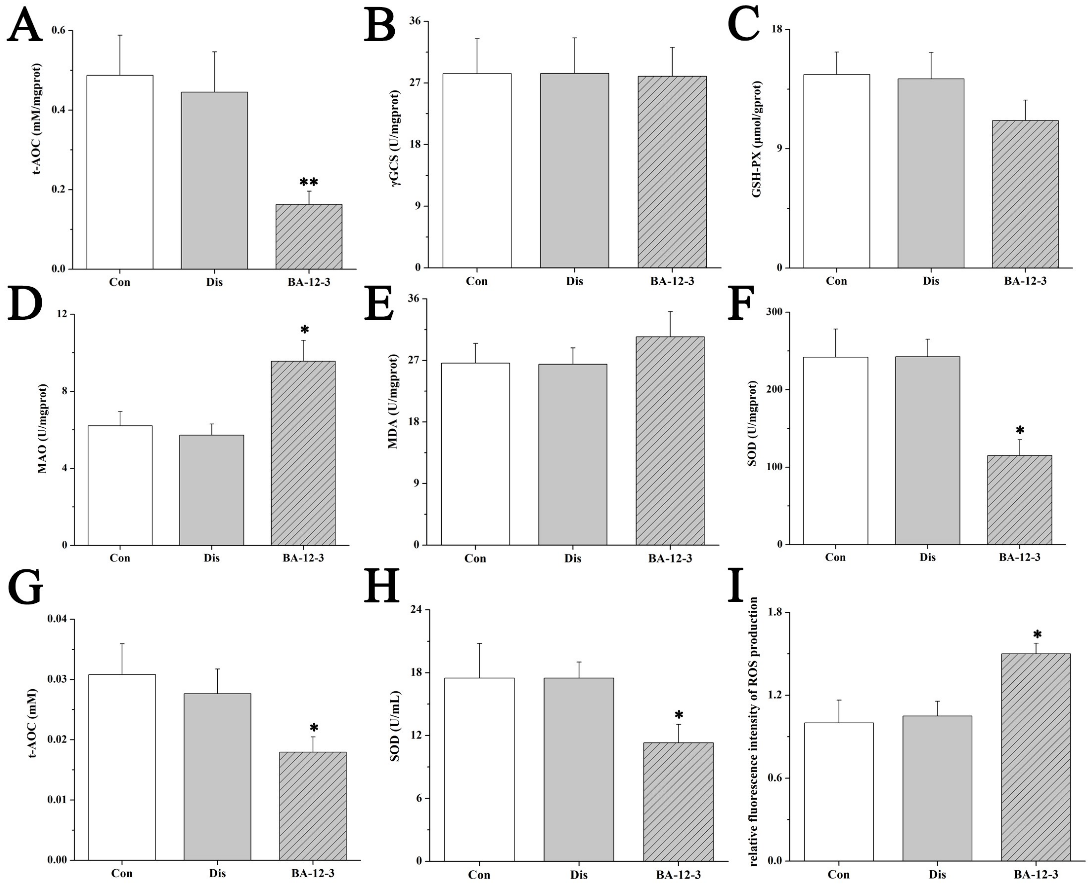

Figure S11 | Effect of BA-12 on REDOX balance. (A-F) Shown are the t-AOC, γGCS, GSH-Px, MAO, MDA, and SOD levels in quail samples, respectively. t-AOC, total antioxidant capacity; γGCS, γ-glutamylcysteine synthetase; GSH-Px, glutathione peroxidase; MAO, monoamine oxidase; MDA, malonadialdehyde; SOD, superoxide dismutase. **(G-I)** Shown are the t-AOC, SOD, ROS levels in cell samples, respectively. ANOVA with the post hoc test was used to calculate the significance of the differences, *, ** and *** represents $p < 0.05$, $p < 0.01$, and $p < 0.001$ compared with the dissolvent group, respectively. All experiments were performed 3 times, and the results are expressed as the mean ± S.D.

12. Prediction pathways regulated by BA-12.

Table S4 | Prediction pathways regulated by BA-12

No.	Term	Count	%	P-Value
1	Metabolic pathways	42	21.4	9.00E-03
2	Pathways in cancer	33	16.8	3.70E-10
3	PI3K-Akt signaling pathway	23	11.7	1.70E-05
4	Proteoglycans in cancer	20	10.2	1.90E-07
5	Ras signaling pathway	18	9.2	2.10E-05

6	Hepatitis B	17	8.7	2.40E-07
7	Osteoclast differentiation	16	8.2	3.60E-07
8	Estrogen signaling pathway	15	7.7	6.20E-08
9	FoxO signaling pathway	15	7.7	2.70E-06
10	Rap1 signaling pathway	15	7.7	4.00E-04
11	Biosynthesis of antibiotics	15	7.7	4.40E-04
12	Prolactin signaling pathway	14	7.1	7.50E-09
13	Insulin resistance	14	7.1	1.30E-06
14	Hepatitis C	14	7.1	1.30E-05
15	Insulin signaling pathway	14	7.1	2.00E-05
16	Tuberculosis	14	7.1	2.60E-04
17	Chemokine signaling pathway	14	7.1	4.20E-04
18	Focal adhesion	14	7.1	1.10E-03
19	Non-small cell lung cancer	13	6.6	4.40E-09
20	Pancreatic cancer	13	6.6	2.60E-08
21	PPAR signaling pathway	13	6.6	3.80E-08
22	Prostate cancer	13	6.6	8.50E-07
23	TNF signaling pathway	13	6.6	6.90E-06
24	Thyroid hormone signaling pathway	13	6.6	1.50E-05
25	Influenza A	13	6.6	7.80E-04
26	cAMP signaling pathway	13	6.6	2.40E-03
27	MAPK signaling pathway	13	6.6	1.60E-02
28	HTLV-I infection	13	6.6	1.60E-02
29	Chronic myeloid leukemia	12	6.1	7.70E-07
30	ErbB signaling pathway	12	6.1	5.30E-06
31	Progesterone-mediated oocyte maturation	12	6.1	5.30E-06
32	T cell receptor signaling pathway	12	6.1	2.00E-05
33	Choline metabolism in cancer	12	6.1	2.20E-05
34	Chagas disease (American trypanosomiasis)	12	6.1	3.00E-05
35	Neurotrophin signaling pathway	12	6.1	1.10E-04
36	Viral carcinogenesis	12	6.1	8.80E-03
37	Colorectal cancer	11	5.6	1.50E-06
38	Glioma	11	5.6	2.30E-06
39	Fc epsilon RI signaling pathway	11	5.6	3.60E-06
40	Toll-like receptor signaling pathway	11	5.6	1.80E-04
41	Toxoplasmosis	11	5.6	2.50E-04
42	Epstein-Barr virus infection	11	5.6	5.70E-04
43	Natural killer cell mediated cytotoxicity	11	5.6	5.70E-04
44	Signaling pathways regulating pluripotency of stem cells	11	5.6	1.60E-03
45	Non-alcoholic fatty liver disease (NAFLD)	11	5.6	2.90E-03
46	Transcriptional misregulation in cancer	11	5.6	5.90E-03
47	Endometrial cancer	10	5.1	2.80E-06
48	Central carbon metabolism in cancer	10	5.1	1.60E-05
49	Adipocytokine signaling pathway	10	5.1	3.40E-05

50	Melanoma	10	5.1	3.80E-05
51	HIF-1 signaling pathway	10	5.1	4.00E-04
52	Sphingolipid signaling pathway	10	5.1	2.00E-03
53	Measles	10	5.1	4.00E-03
54	Jak-STAT signaling pathway	10	5.1	7.00E-03
55	Acute myeloid leukemia	9	4.6	4.30E-05
56	Renal cell carcinoma	9	4.6	1.40E-04
57	Epithelial cell signaling in Helicobacter pylori infection	9	4.6	1.60E-04
58	Complement and coagulation cascades	9	4.6	2.00E-04
59	Fc gamma R-mediated phagocytosis	9	4.6	7.60E-04
60	Small cell lung cancer	9	4.6	8.20E-04
61	GnRH signaling pathway	9	4.6	1.30E-03
62	Carbon metabolism	9	4.6	5.00E-03
63	Bladder cancer	8	4.1	4.00E-05
64	VEGF signaling pathway	8	4.1	5.30E-04
65	B cell receptor signaling pathway	8	4.1	1.10E-03
66	Adherens junction	8	4.1	1.30E-03
67	Chemical carcinogenesis	8	4.1	2.70E-03
68	AMPK signaling pathway	8	4.1	2.60E-02
69	Platelet activation	8	4.1	3.30E-02
70	Apoptosis	7	3.6	3.20E-03
71	Metabolism of xenobiotics by cytochrome P450	7	3.6	7.70E-03
72	Pertussis	7	3.6	8.20E-03
73	Inflammatory mediator regulation of TRP channels	7	3.6	2.80E-02
74	Serotonergic synapse	7	3.6	4.70E-02
75	Cholinergic synapse	7	3.6	4.70E-02
76	Renin-angiotensin system	6	3.1	1.60E-04
77	Type II diabetes mellitus	6	3.1	5.10E-03
78	Arginine and proline metabolism	6	3.1	6.10E-03
79	Regulation of lipolysis in adipocytes	6	3.1	9.90E-03
80	NOD-like receptor signaling pathway	6	3.1	9.90E-03
81	mTOR signaling pathway	6	3.1	1.10E-02
82	Arachidonic acid metabolism	6	3.1	1.40E-02
83	Glycolysis / Gluconeogenesis	6	3.1	2.00E-02
84	Drug metabolism - cytochrome P450	6	3.1	2.20E-02
85	Thyroid cancer	5	2.6	4.40E-03
86	Aldosterone-regulated sodium reabsorption	5	2.6	1.30E-02
87	Steroid hormone biosynthesis	5	2.6	4.70E-02
88	Dorso-ventral axis formation	4	2	2.50E-02
89	Fructose and mannose metabolism	4	2	3.90E-02
90	Tyrosine metabolism	4	2	4.80E-02
