# Supplementary figures and images for: BA-12 Inhibits Angiogenesis via Glutathione Metabolism Activation
Source: Int J Mol Sci. 2019 Aug 20;20(16):4062. doi: 10.3390/ijms20164062 (PMC6720627; doi:10.3390/ijms20164062)

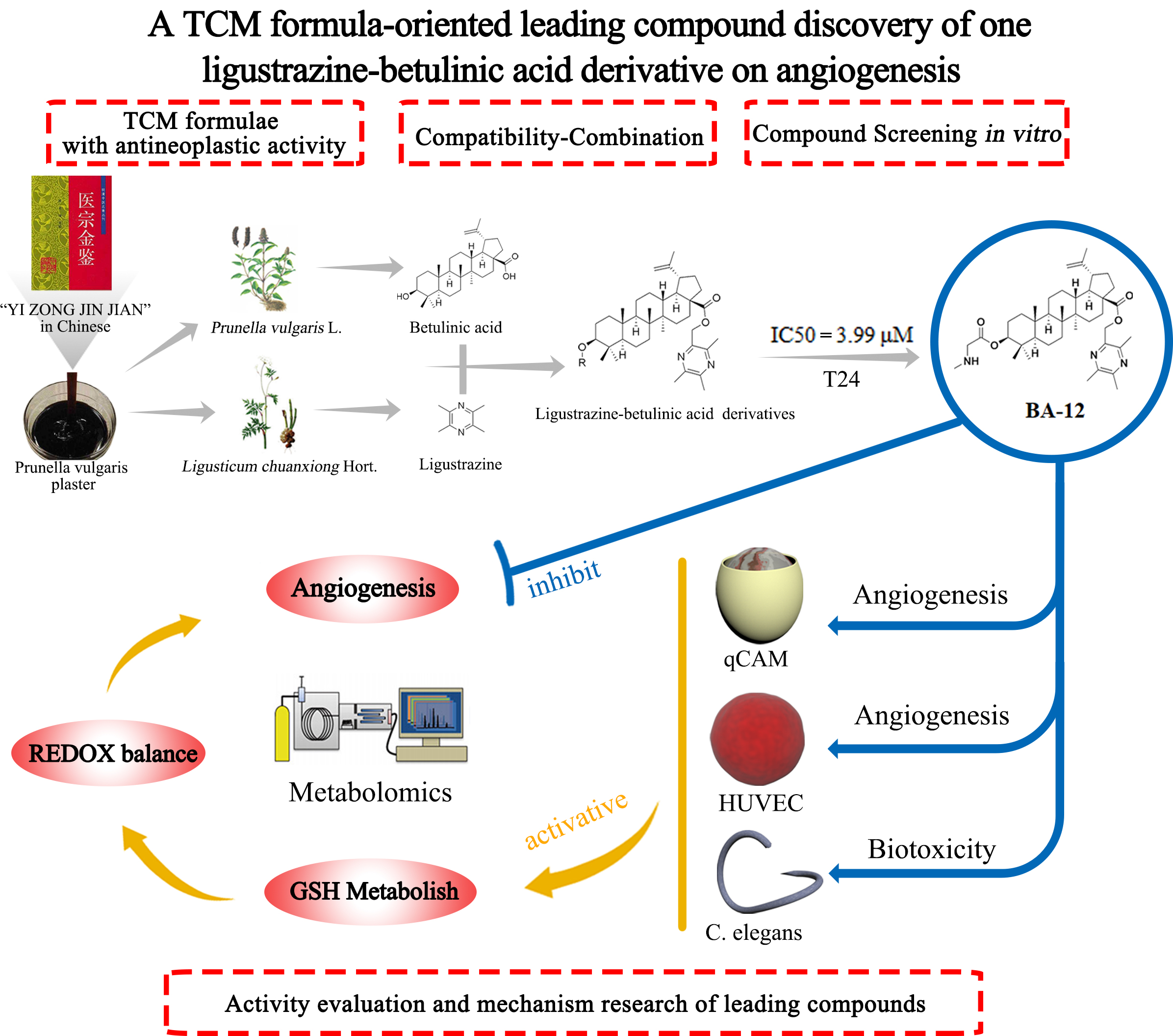

Supplement: Supplementary file 1 [file ijms-20-04062-s001.zip › FIGURE 1.tif]

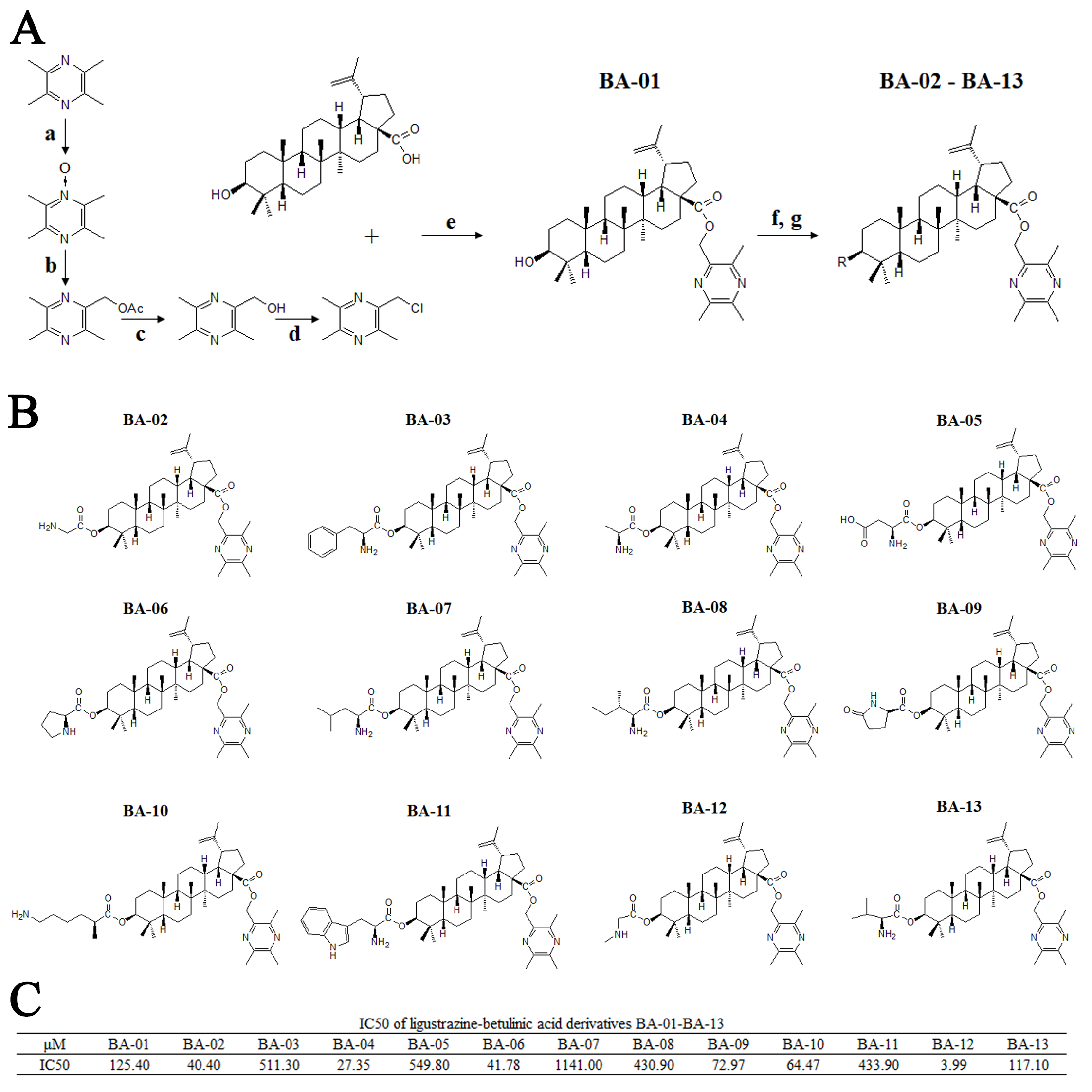

Supplement: Supplementary file 1 [file ijms-20-04062-s001.zip › FIGURE 2.tif]

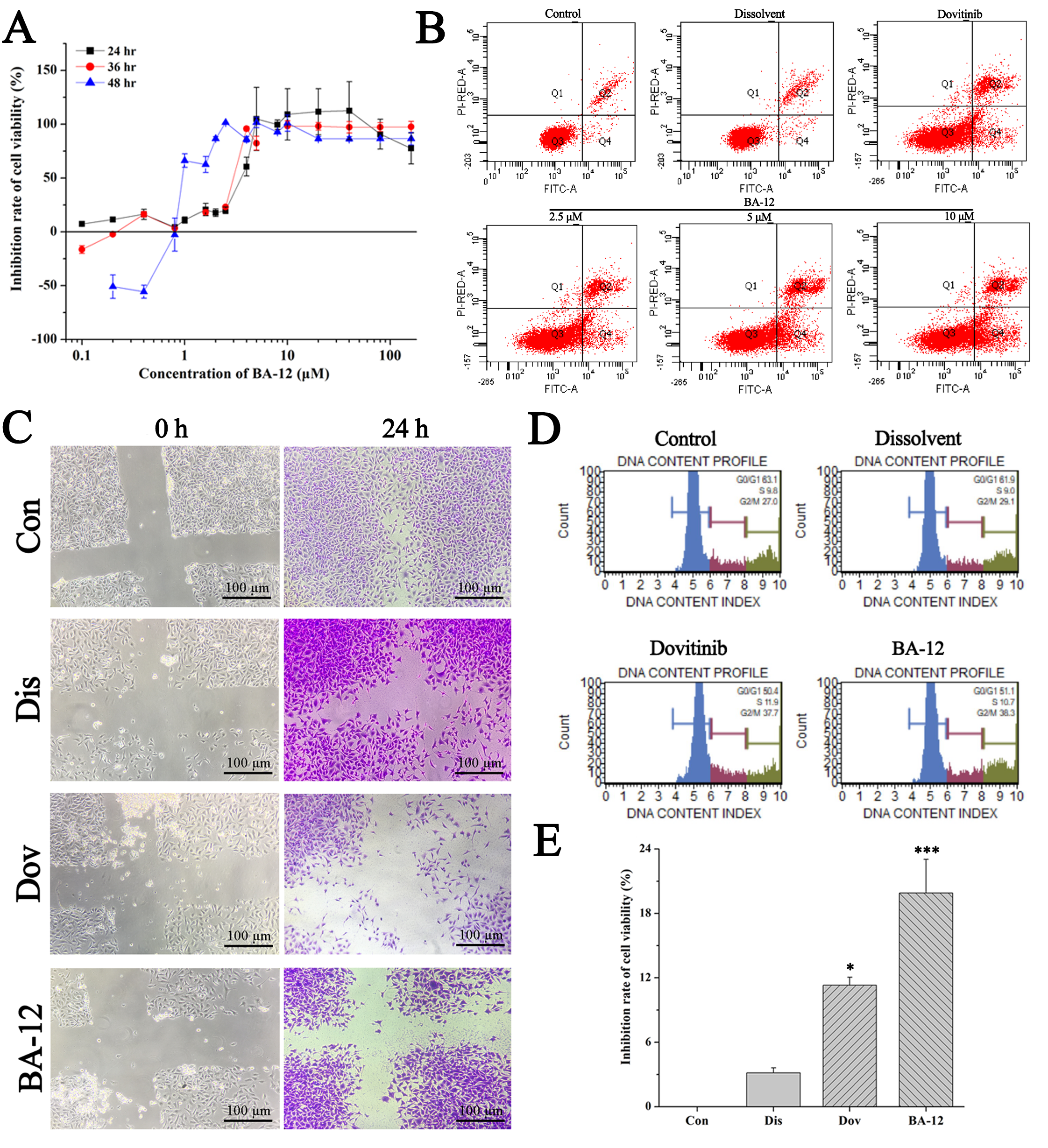

Supplement: Supplementary file 1 [file ijms-20-04062-s001.zip › FIGURE 3.tif]

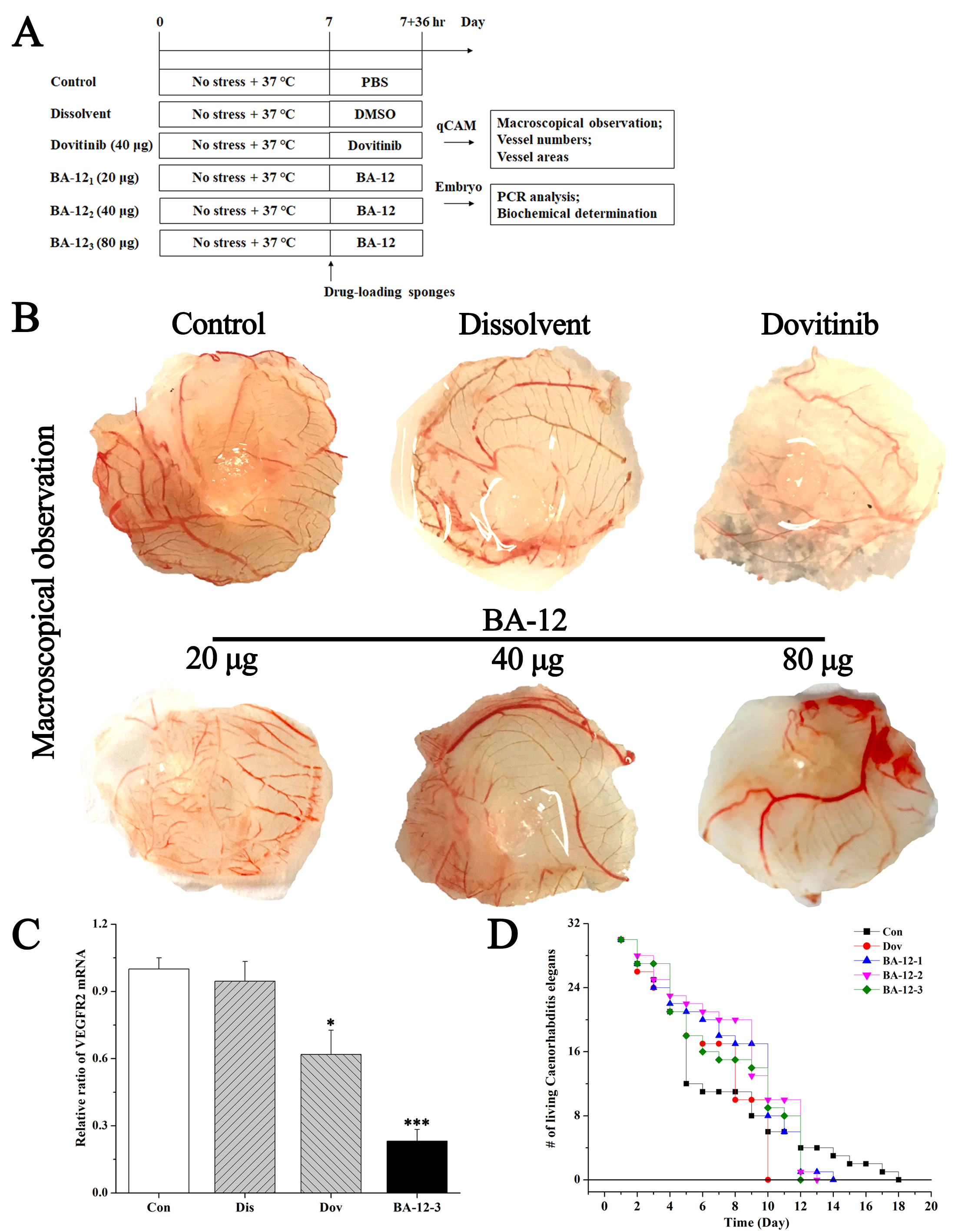

Supplement: Supplementary file 1 [file ijms-20-04062-s001.zip › FIGURE 4.tif]

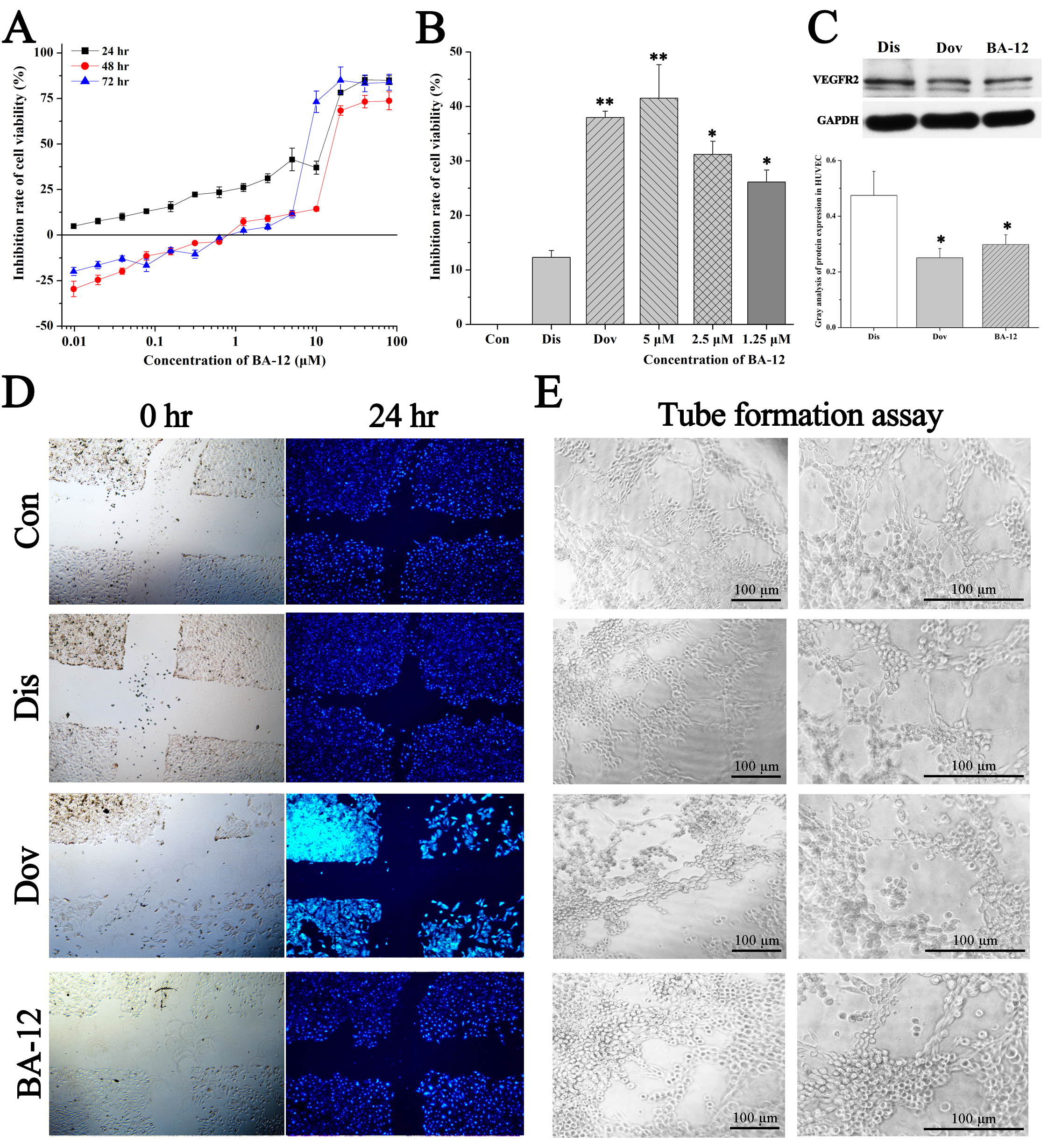

Supplement: Supplementary file 1 [file ijms-20-04062-s001.zip › FIGURE 5.tif]

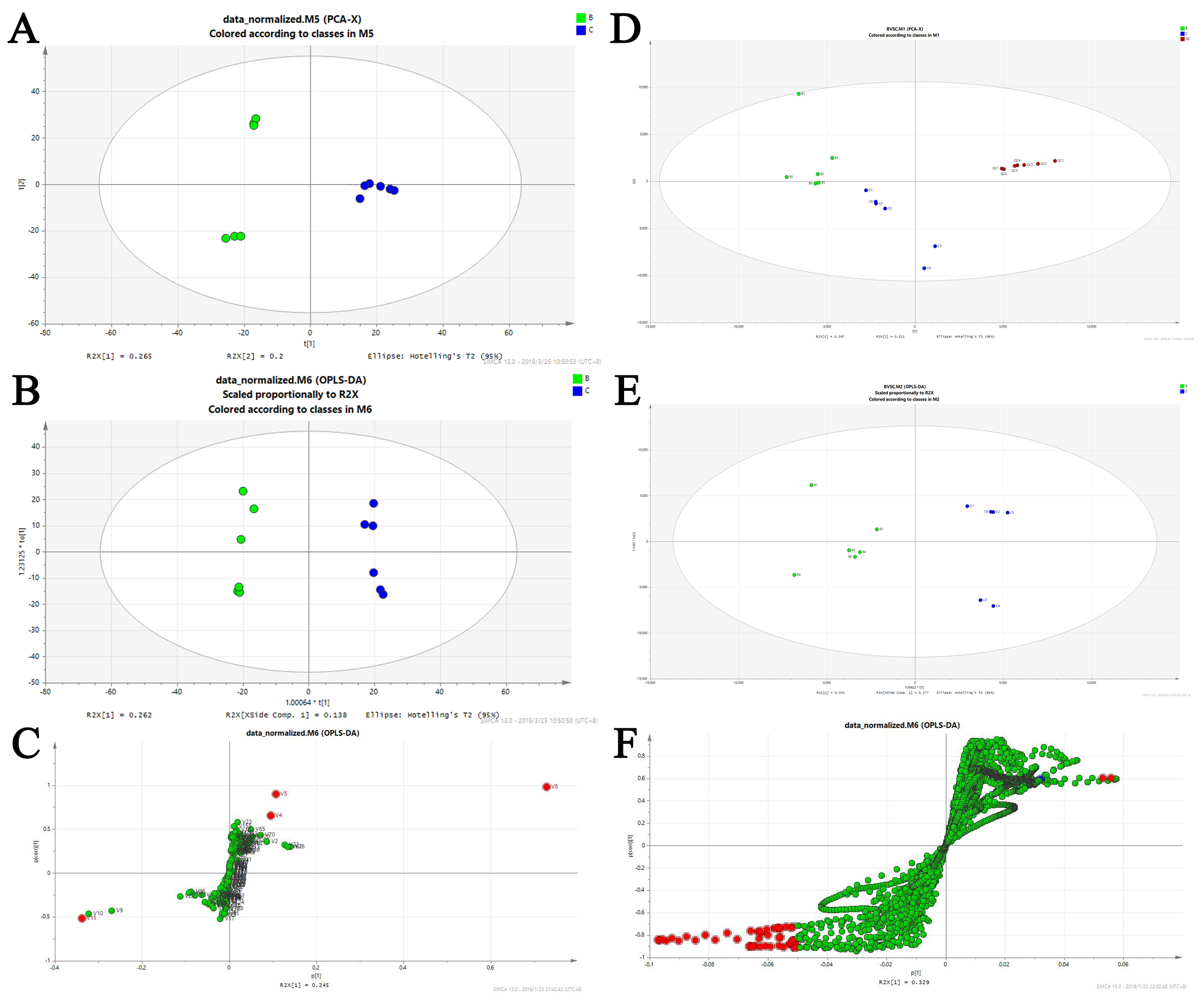

Supplement: Supplementary file 1 [file ijms-20-04062-s001.zip › FIGURE 6.tif]

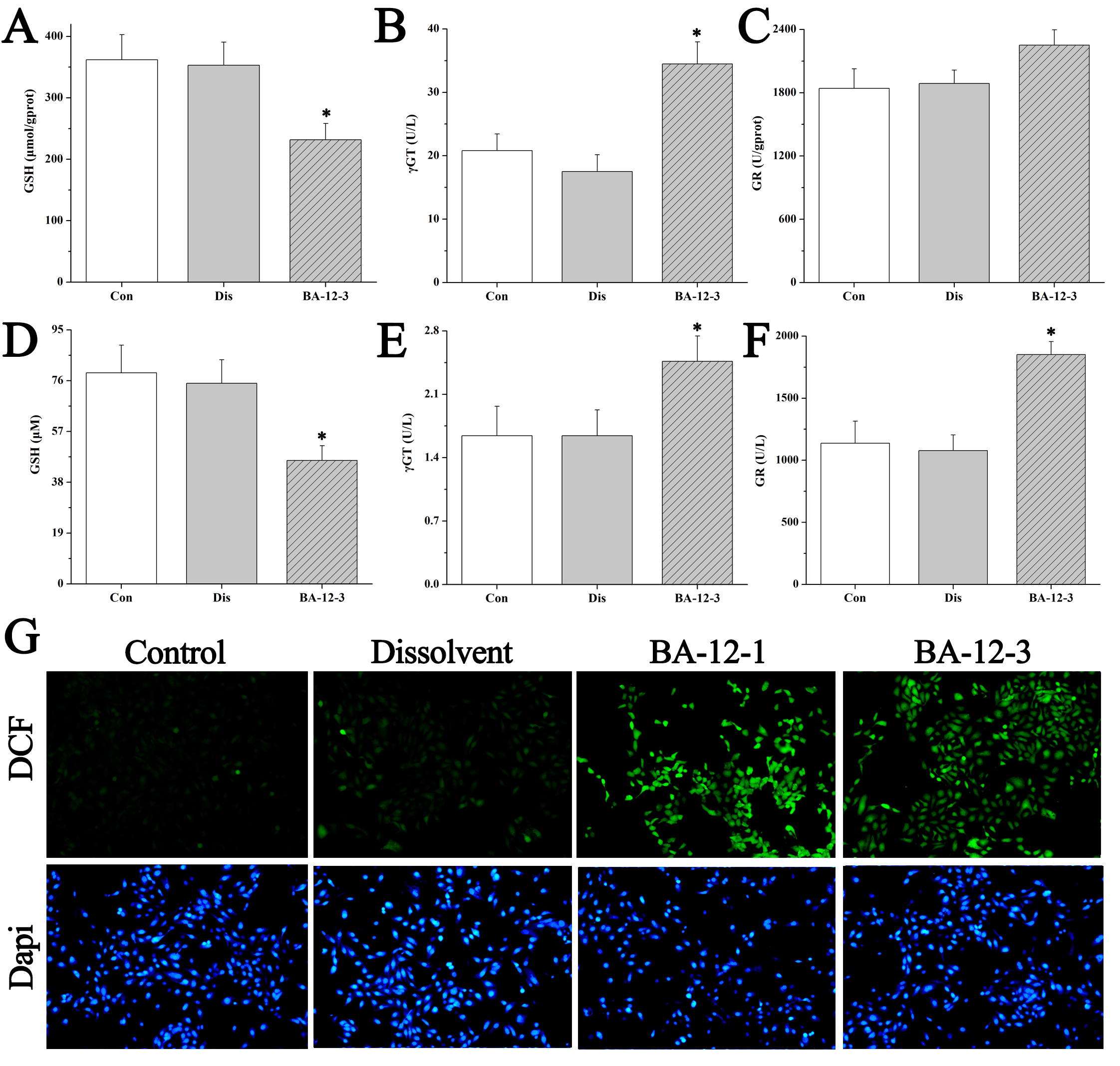

Supplement: Supplementary file 1 [file ijms-20-04062-s001.zip › FIGURE 7.tif]

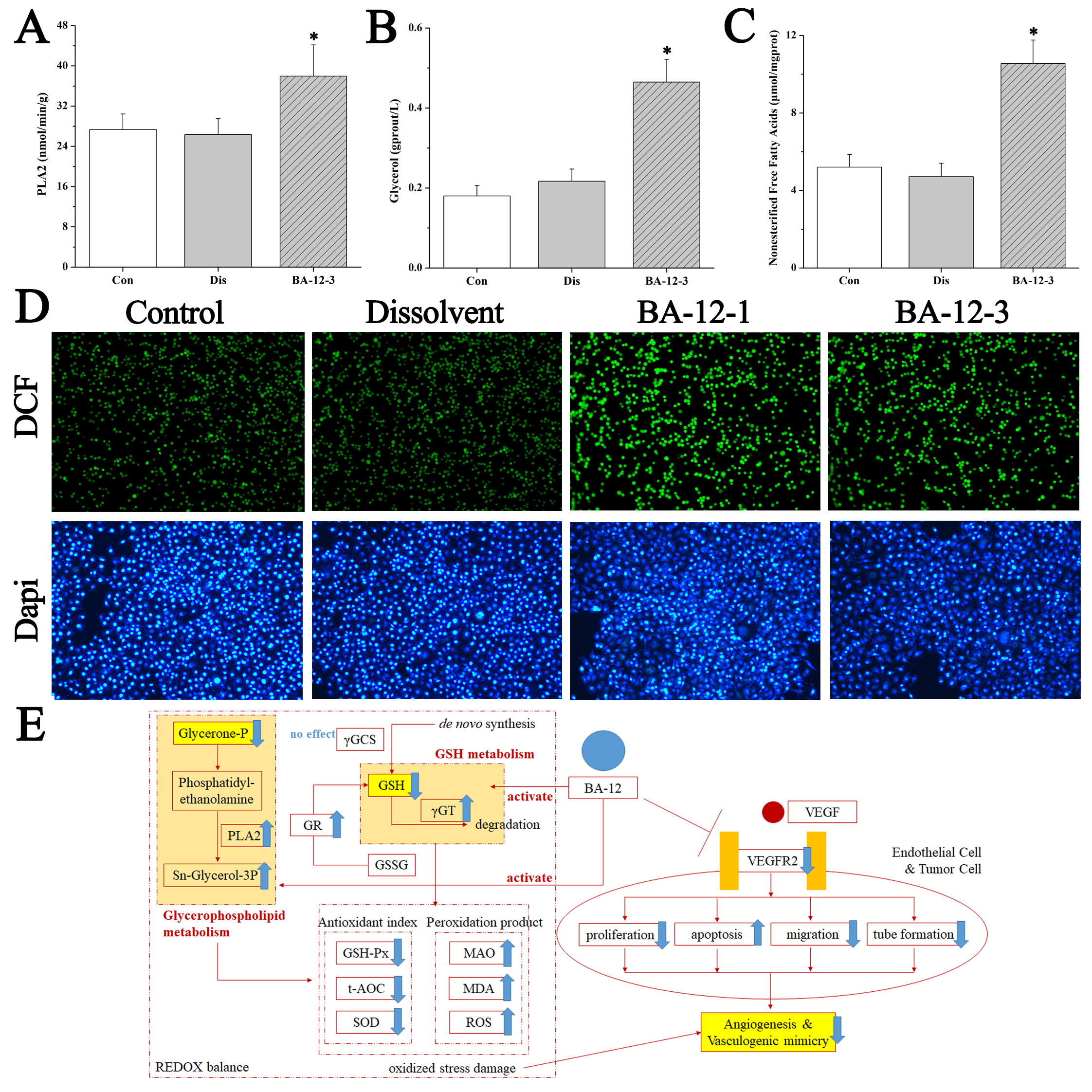

Supplement: Supplementary file 1 [file ijms-20-04062-s001.zip › FIGURE 8.tif]
